# Rapidly self-healing electronic skin for machine learning–assisted physiological and movement evaluation

**DOI:** 10.1126/sciadv.ads1301

**Published:** 2025-02-12

**Authors:** Yongju Lee, Xinyu Tian, Jaewon Park, Dong Hyun Nam, Zhuohong Wu, Hyojeong Choi, Juhwan Kim, Dong-Wook Park, Keren Zhou, Sang Won Lee, Tanveer A. Tabish, Xuanbing Cheng, Sam Emaminejad, Tae-Woo Lee, Hyeok Kim, Ali Khademhosseini, Yangzhi Zhu

**Affiliations:** ^1^ Terasaki Institute for Biomedical Innovation, Los Angeles, CA 91367, USA.; ^2^ School of Electrical and Computer Engineering, Center for Smart Sensor System of Seoul (CS4), University of Seoul, Seoul 02504, Republic of Korea.; ^3^ Department of Nanoengineering, University of California, San Diego, La Jolla, CA 92093, USA.; ^4^ Division of Cardiovascular Medicine, Radcliffe Department of Medicine, British Heart Foundation (BHF) Centre of Research Excellence, University of Oxford, Headington, Oxford OX3 7BN, UK.; ^5^ Department of Electrical and Computer Engineering and Department of Bioengineering, University of California Los Angeles, Los Angeles, CA 90095, USA.; ^6^ Department of Materials Science and Engineering, Seoul National University, Seoul 08826, Republic of Korea.

## Abstract

Emerging electronic skins (E-Skins) offer continuous, real-time electrophysiological monitoring. However, daily mechanical scratches compromise their functionality, underscoring urgent need for self-healing E-Skins resistant to mechanical damage. Current materials have slow recovery times, impeding reliable signal measurement. The inability to heal within 1 minute is a major barrier to commercialization. A composition achieving 80% recovery within 1 minute has not yet been reported. Here, we present a rapidly self-healing E-Skin tailored for real-time monitoring of physical and physiological bioinformation. The E-Skin recovers more than 80% of its functionality within 10 seconds after physical damage, without the need of external stimuli. It consistently maintains reliable biometric assessment, even in extreme environments such as underwater or at various temperatures. Demonstrating its potential for efficient health assessment, the E-Skin achieves an accuracy exceeding 95%, excelling in wearable muscle strength analytics and on-site AI-driven fatigue identification. This study accelerates the advancement of E-Skin through rapid self-healing capabilities.

## Teaser

Self-healing E-Skin achieves 80% recovery in 10 s, enabling real-time monitoring of health assessments.

## INTRODUCTION

Electronic skins (E-Skins) represent a transformative technology that integrates seamlessly with the paradigms of telehealth, personalized health care, and precision medicine (*1*, *2*). E-Skins facilitate the active and continuous monitoring of physical, physiological, and chemical biosignatures related to fatigue while maintaining comfort and minimizing disruption to daily activities, thus significantly enhancing the capacity for self-monitoring and substantially promoting the overall quality of health care delivery (*3*–*5*). Specifically, real-time wearable electrophysiological devices are widely used to monitor muscle conditions in a noninvasive manner (*6*, *7*). This enables the assessment of muscle fatigue and the evaluation of postoperative muscle rehabilitation as well as the facilitation of proactive and personalized interventions tailored to individual physiological responses, thus ensuring both safety and performance.

The field has advanced over the past 20 years, yet persistent challenges remain unresolved. Critical issues, whether related to materials or devices, continue to hinder progress. Unexpected mechanical damage caused by repeated wear and tear and accidental cutting or scratching is the leading cause of device failure when undertaking labor-intensive and repetitive tasks, both indoors and outdoors. So far, achieving the requisite performance within 1 min after mechanical damage has proven difficult, posing a notable material challenge that remains unmet. Consequently, the 1-min performance threshold necessary for commercialization has not been attained. In addition, there is also the hurdle for E-skin technology to be effectively integrated into daily life.

Here, we report an E-Skin capable of rapidly self-repairing after unexpected internal or external mechanical damage, recovering critical functions with a high recovery rate of 80% within 10 s and remaining operable. The rapidly self-healing E-Skin is designed to offer noninvasive electrophysiological monitoring and machine learning–assisted fatigue evaluation during daily routines. Because of its high mechanical-electrical conversion efficiency, the E-Skin can also accurately track diverse human motions without requiring an external energy supply. To effectively simulate and evaluate the practical application potential of the E-Skin under realistic conditions, experimental parameters were selected to encompass a broad spectrum of environments. These parameters were deliberately chosen to mirror real-world usage scenarios, such as extreme temperatures and varying humidity levels, ensuring that the self-healing capabilities remain reliable across diverse operational conditions. Moreover, we develop an on-site AI-driven analytical tool capable of identifying and classifying muscle fatigue with more than 95% accuracy. In addition, the system features a personalized graphical user interface, enabling comprehensive real-time biometric interpretation.

## RESULTS

### Structural design, self-healing capability, and flexibility of the E-Skin

**Fig. 1.**
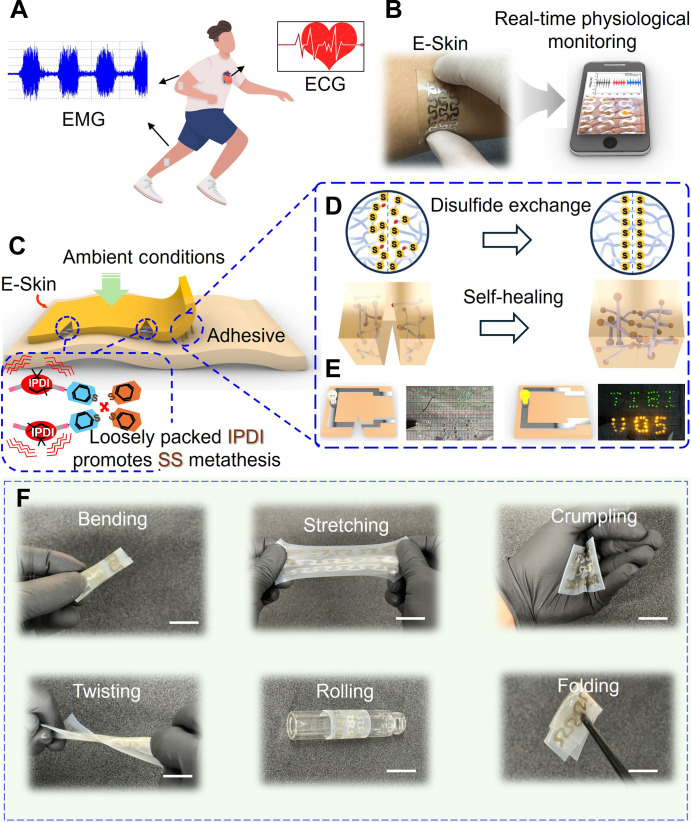
Structural design, self-healing capability, and mechanical flexibility of the E-Skin. (**A**) Schematic illustration of the E-Skin designed for continuous, noninvasive monitoring of electrophysiological (sEMG and ECG) and joint movements. (**B**) Digital image showing the E-Skin conformally applied to the epidermis. (**C**) Schematic representation of the self-healing mechanism of the E-Skin, highlighting the incorporation of IPDI into the TPU matrix, where it interacts with disulfide bonds (SS) to enhance chain mobility and promote self-healing, emphasizing the role of IPDI in maintaining the flexibility and integrity of the rapid self-healable E-Skin. (**D**) Schematic depicting the self-healing capability through disulfide exchange. (**E**) Schematics and images demonstrate the restoration of conductivity of the E-Skin after being completely cut, as evidenced by successful powering of LED arrays. (**F**) Photographic images illustrating the mechanical performance and durability of the E-Skin. Scale bars, 1 cm.

As illustrated conceptually in Fig. 1A, the E-Skin provides wearable electrophysiological monitoring, including surface electromyography (sEMG), electrocardiography (ECG), and self-powered motion tracking. This E-Skin adheres seamlessly and conformably to the epidermis, enabling real-time results display on mobile devices (Fig. 1B). The primary material used is fibrous thermoplastic polyurethane (TPU), chosen for its softness, excellent breathability (water/air permeability), and self-protective abilities against adverse loading events (*8*–*10*). Specifically, TPU is composed of both soft and hard segments, with the soft segments providing flexibility and the hard segments contributing mechanical strength. Its thermoplastic nature enables high chain mobility at elevated temperatures, facilitating bond reformation and supporting efficient self-healing. In addition, TPU’s robust hydrogen bonds facilitate the rapid reconnection of damaged areas without requiring external stimuli, making it an ideal material for balancing durability and flexibility while promoting fast self-repair. Its excellent biocompatibility further ensures safety for extended skin contact (*11*–*13*). Polytetramethylene ether glycol (PTMEG) soft segments were further incorporated to enhance its softness, achieving a tissue-like consistency with a Young’s modulus of approximately 0.1 MPa (*14*, *15*). The self-healing capability is essential to ensure that the E-Skin retains its functionality despite continuous mechanical stress from actions such as flipping, folding, and stretching, as well as potential user mishandling, such as accidental cuts or scratches during labor-intensive and repetitive tasks. To accomplish this, we used TPU as the backbone for the E-Skin and incorporated *bis*(4-hydroxyphenyl)disulfide into the matrix to promote the formation of intramolecular disulfide bonds (fig. S1) (*16*, *17*). We incorporated isophorone diisocyanate (IPDI) into the TPU matrix. The asymmetric alicyclic structure of IPDI significantly enhances the flexibility and mobility of the polymer chains, which is essential for effective self-healing. IPDI facilitates the disulfide metathesis reaction within the TPU matrix, enabling the reversible breaking and reformation of disulfide bonds autonomously. The biocompatibility of the IPDI-based TPU is clarified in note S1 (*18*, *19*). This reversible bonding mechanism is fundamental to the self-healing process, allowing it to occur without requiring heat or other external stimuli (Fig. 1C and note S2). These disulfide (R─S─S─R′) bonds readily form through redox reactions between thiol (−SH) groups in cysteine amino acids, even under ambient conditions, due to their very low redox potentials (Fig. 1D and fig. S2) (*20*, *21*). A detailed summary of the material optimization process is provided in note S3 and figs. S3 and S4.

The silver nanowire (AgNW) conductive patterns were printed onto the substrate to ensure sufficient conductivity (*22*, *23*). The justification for using AgNWs as the electrode material in this study is detailed in note S4 and table S1 [(*24*–*27*)] and fig. S5. As shown in Fig. 1E and movies S1, the E-Skin demonstrated its ability to fully restore mechanical and electrical properties after complete severance, evidenced by a standalone light-emitting diode (LED) that resumed illumination within seconds. This rapid recovery of electrical conduction pathways after mechanical damage is attributed to the dynamic reconfiguration of nanostructured conductive networks. These networks comprise a one-dimensional (1D) AgNW network interfaced with a cross-linked, self-healing polymer matrix. The excellent durability and mechanical flexibility of the E-Skin allowed it to adapt to various dynamic deformation conditions (i.e., bending, stretching, crumpling, twisting, rolling, and folding) that can occur during device handling, cleaning, storage, and fitting without mechanical failure (Fig. 1F). Also, the patch thickness could be further decreased via tuning the parameters (i.e., speed and duration) of the spin coating (fig. S6). To further evaluate the durability of the E-Skin under repeated mechanical deformation, a bending test was performed with a radius of 5 cm for up to 50,000 cycles (fig. S7). Only a 10% increase in resistance was observed, suggesting its structural and functional integrity when subjected to repeated mechanical stress.

### Mechanical performance, stretchability, and self-healing properties of the E-Skin

**Fig. 2.**
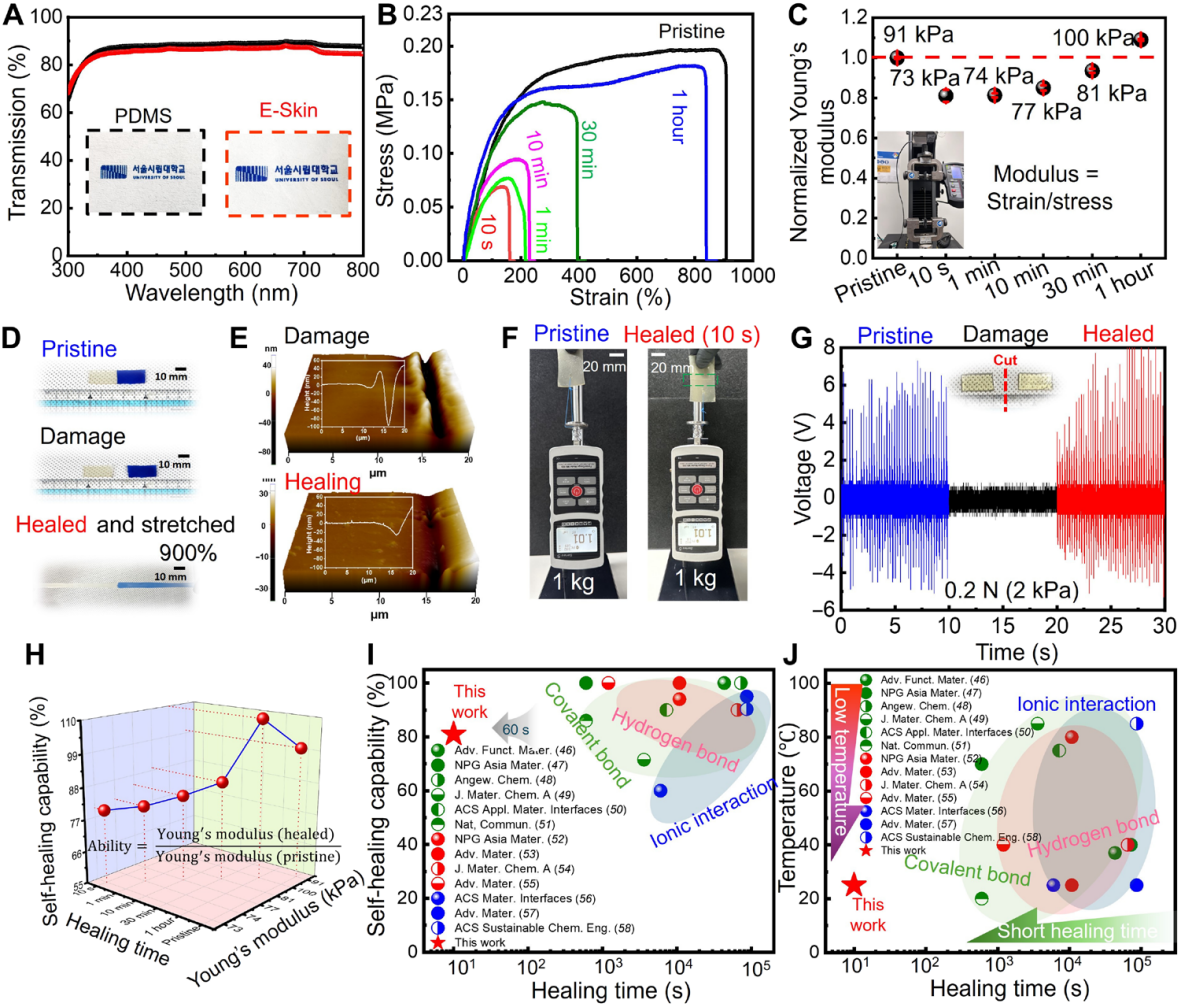
Optical properties, stretchability, and self-healing characteristics of the E-Skin. (**A**) Wavelength-dependent transmittance and a corresponding photograph comparing the E-Skin with commonly used PDMS. (**B**) Stress-strain profile of the E-Skin throughout the healing process following a complete cut. (**C**) Changes in Young’s modulus observed during the healing process. (**D**) Photographs illustrating the self-healing process of two rectangular E-Skin devices under ambient conditions, without external stimulation, highlighting its ability to stretch up to 900% post-healing. (**E**) Atomic force microscopy (AFM) measurements capturing the healing progression. (**F**) Photographic images showing the damaged E-Skin’s capacity to support a 1 kg of weight within 10-s post-cut, without external stimulation. (**G**) E-skin sensing test under 0.2-N external stimulation. (**H**) A 3D graph showing the self-healing capability and Young’s modulus of the E-Skin post-cut. (**I** and **J**) Comparative analysis of the E-Skin developed in this work with previously reported self-healing devices (*46*–*58*), evaluating healing time, self-healing ability, and temperature. The additional comparison of the E-Skin with existing devices is summarized in table S2.

To characterize the mechanical capabilities of the E-Skin, we plotted the stress-strain curve (Fig. 2A) and calculated the Young’s modulus (Fig. 2B) after complete severance. As shown in Fig. 2C, the healed E-Skin demonstrated great elasticity, sustaining up to 900% stretchability. The Young’s modulus exhibited superior mechanical properties 1 hour post–self-healing compared to its original state. This enhancement is likely due to the increased formation of disulfide bonds, which bolster the structural integrity of the E-Skin patch. During the healing process, microstructured changes occur, such as the densification and alignment of polymer chains, leading to improved mechanical properties. These changes contribute to the observed increase in Young’s modulus by enhancing the elasticity and strength of the E-Skin. Atomic force microscopy validated the successful healing process (Fig. 2D). Moreover, the healed E-Skin preserved its structural integrity while supporting a load of 1 kg, which is 1500 times its own weight, without any signs of failure (Fig. 2E). Post-healing functionality was verified by attaching the E-Skin to the body, where it effectively continued to detect force through the triboelectric effect (Fig. 2F and movie S2). The self-healing ability can be defined as

$$\text{Self healing ability}=\frac{\text{Young}’s\;\text{modulus}\;\left(\text{healed}\;E–\text{Skin}\right)}{\text{Young}’s\;\text{modulus}\;\left(\text{pristine}\;E–\text{Skin}\right)}$$

Young’s modulus was ascertained by examining the slope of the stress-strain curve, as shown in Fig. 2A. (*28*) The self-healing capacity of the E-Skin was restored to approximately 80% within just 10 s (Fig. 2H). Movie S3 demonstrates the dynamic self-healing process of the E-Skin under ambient conditions after being completely cut using polydimethylsiloxane (PDMS), a widely used E-Skin material, as a comparative benchmark.

As mentioned above, incorporating IPDI with an asymmetric alicyclic structure into the TPU matrix enhances chain mobility, thereby promoting disulfide metathesis for self-healing while preserving mechanical robustness (*29*, *30*). The asymmetric structure of IPDI ensures sufficient chain mobility, facilitating self-healing even at low temperatures. These bonds can be activated at room temperature without the need for additional heat or external stimuli and are also responsive at intermediate temperatures (60°–90°C), enabling efficient self-healing across a wide range of temperatures, from low and high. Figure S8 presents the temperature-dependent kinetics of the self-healing process of the E-Skin. Elevated temperatures significantly accelerate this process by enhancing interactions among the polymer chains within the E-Skin. Specifically, the complete healing time decreased to about 7 min at 40°C, a temperature comparable to the skin surface during outdoor operations. In addition, fig. S9 evaluates the long-term durability and repeatability of the E-Skin through repetitive damaging (cutting)–healing cycles at various temperatures (30 cycles at 25°C, 50 cycles at 40°C, and 100 cycles at 60°C).

The findings demonstrate minimal variation in the self-healing ability of the E-Skin even after extended use. Movie S4 showcases the E-Skin’s practical application potential across various temperatures (−3°, 25°, 36°, and 50°C) and underwater, underscoring its waterproof properties due to the protective PDMS layer. This waterproof capability was further corroborated by pressure-sensing performance tests under different humidity conditions (40, 60, and 80%), as depicted in fig. S10. We assessed the self-healing performance under varying humidity levels of 40, 60, and 80%, representing a range of indoor and outdoor environmental conditions, ranging from relatively dry to highly humid settings (fig. S11). A comprehensive explanation of the criteria used to establish these aforementioned experimental conditions for evaluating self-healing performance is provided in note S5.

We further evaluated the changes in the physical and electrical properties of the E-Skin following repeated self-healing cycles. As shown in fig. S12, the E-Skin was subjected to up to 40 cycles of cutting and self-healing at 10-s intervals, with continuous monitoring of its electrical output during touch tests. The results indicate the reliability of the rapid self-healing capability of the E-Skin. In addition, we conducted up to 50 self-healing tests, measuring conductivity after each cycle (fig. S13). The results indicate negligible degradation, highlighting the E-Skin’s superior durability, even after multiple cycles of cutting and self-healing. Figure 2I highlights the self-healing ability of the E-Skin over time, while Fig. 2J illustrates the correlation between self-healing ability and temperature. A comprehensive comparison has also been summarized in table S2. Moreover, the presence of sweat does not influence the self-healing capabilities, as consistent electrical output is maintained from the self-healed E-Skin patch on sweating skin, comparable to that of the pristine E-Skin patch (fig. S14).

### Whole-body monitoring of physiological signals and joint movements

Excessive work-related physiological demands can adversely affect safety and productivity by diminishing workers’ well-being, attentiveness, motivation, and manual labor capacity (*31*). Thus, it is imperative to assess their physiological conditions using comfortable, nonintrusive monitoring tools designed for everyday use, which are essential for any ergonomics study.

**Fig. 3.**
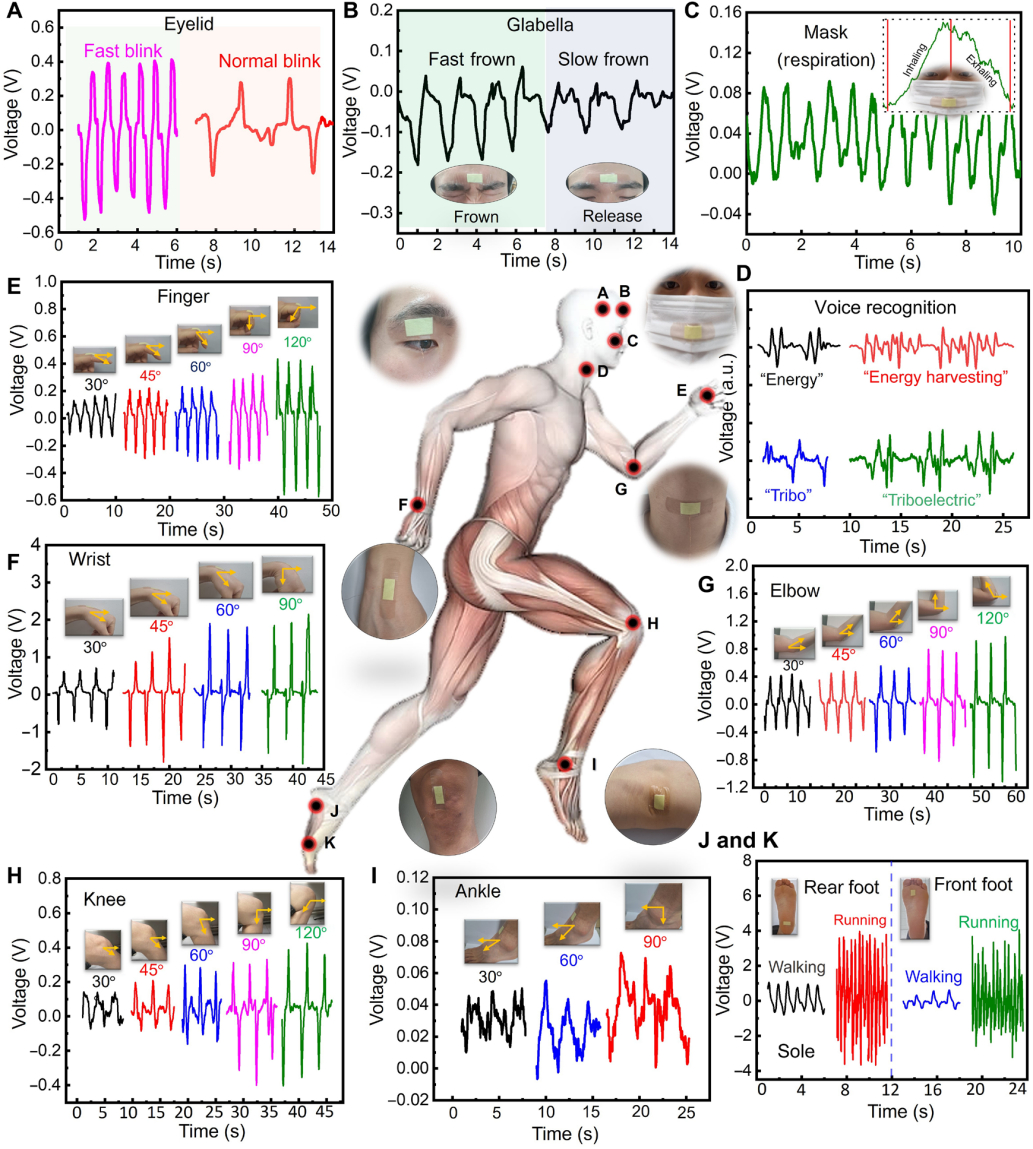
Comprehensive whole-body monitoring of physiological signals and joint movements using E-Skin. (**A**) Monitoring blinking behavior by attaching the E-Skin to the eyelid. (**B**) Voltage response to frowning, with the E-Skin applied on the forehead. (**C**) Continuous monitoring of oral breathing using the E-Skin under a dust mask; the inset shows the voltage signal during a single inhalation-exhalation cycle. (**D**) Sound and speech recognition via E-Skin mounted on a volunteer’s throat to detect vocal cord vibrations. (**E**) Detection of finger-bending angles. (**F**) Detection of wrist-bending angles. (**G**) Detection of arm flexion. (**H**) Measurement of leg-swing angles. (**I**) Detection of leg flexion. (**J** and **K**) Monitoring of foot movements with the E-Skin attached to the corresponding limb.

Owning to its exceptional pressure sensitivity and conformal capability, our E-Skin enables real-time, noninvasive, and self-powered monitoring of various physiological signals. The triboelectric effect offers a robust strategy for powering E-Skin applications (*32*–*34*). Detailed information of the self-healing mechanism can be found in note S6 and fig. S15. For accurate monitoring of subtle signals, it is essential for the E-Skin to establish conformal and intimate contact with the epidermis. This was achieved by securely attaching the E-Skin to the epidermis using biocompatible medical adhesive (Fig. 3). In addition, the application of medical adhesive has a minimal impact on the self-healing performance (fig. S16). Facial expressions—including ocular blinking, frowning, and smiling—are primary channels for conveying human emotions and external communication. The E-Skin can capture minute muscle movements from microexpressions through variations in voltage signals. Specifically, subtle voltage signals induced by blinking were detected when the E-Skin was mounted on an eyelid (Fig. 3A). In addition, the E-Skin can differentiate between normal and rapid blinking based on variations in frequency and amplitude.

To achieve accurate monitoring, it is essential for the E-Skin to maintain conformal and intimate contact with the epidermis. This was accomplished by securely attaching the E-Skin to the skin using biocompatible medical bandages (Fig. 3). Facial expressions such as blinking, frowning, and smiling are fundamental for conveying human emotions and serve as primary external communication channels. The minute muscle movements associated with microexpressions can be effectively captured through variations in voltage signals. Specifically, subtle voltage changes induced by blinking were detected by affixing the E-Skin to an eyelid (Fig. 3A). Furthermore, distinctions in the frequency and amplitude of regular versus rapid blinking were also discernible.

Attaching the E-Skin to the forehead allowed for monitoring consistent and reproducible voltage signals during alternating regular and frowning movements (Fig. 3B). Respiration, detectable through the flow of breath or the expansion and contraction of the chest and abdomen during inhalation and exhalation, is a vital sign for assessing users’ health. When positioned on the vent of a conventional mask, the E-Skin successfully identified voltage signal changes associated with repeated oral breathing (Fig. 3C).

Upon conformal attachment to the throat, the E-Skin effectively recognized distinct words and phrases such as “energy,” “energy harvesting”, “tribo,” and “triboelectric”. This demonstrates the voice recognition capabilities (Fig. 3D and fig. S17). Each word was recorded three times, consistently generating similar voltage signal responses, indicating high repeatability for voice recognition. This feature could be instrumental in rehabilitating the speech abilities of users with damaged vocal cords by training them to control their throat muscle movements through remote human-machine interaction and control. In addition, the E-Skin demonstrates a robust response to subtle skin-level stimuli and can accurately track small joint movements, including the knuckle, elbow, knee, and ankle. For instance, when attached to the lateral knuckle of the index finger, the E-Skin recorded voltage responses during multiple bending-releasing cycles, with bending angles ranging from 30° to 120° (Fig. 3E).

The E-Skin effectively detected motion angles across various joints, including the wrist (Fig. 3F), elbow (Fig. 3G), knee (Fig. 3H), and ankle (Fig. 3I). When applied to these joints and subjected to different degrees of bending and straightening, the E-Skin produced consistent and reproducible voltage signals corresponding to the joint angles, demonstrating its stability and reliability. Minor variations in the output frequencies of the voltage signals were observed, likely due to slight inconsistencies in movement patterns. The increased voltage output with larger bending angles is attributed to the expanded contact area between the E-Skin and the joints. The accurate recognition of joint motion angles facilitated by the E-Skin holds significant potential for real-time remote operation of robotic movements, thereby enhancing human-machine interaction capabilities.

The human movement state is a critical indicator of work intensity, which can be effectively monitored by placing the E-Skin on the ankle. Figure 3 (J and K) shows that attaching the E-Skin to either the back or front of the foot results in notable variations in the amplitude and frequency of the voltage signals, enabling the differentiation between walking and running. These examples illustrate that diverse physiological characteristics and movements can be translated into readable, quantifiable, real-time voltage signals via the E-Skin, thus offering a promising approach for comprehensive electrophysiological and motion monitoring. Therefore, the E-Skin shows great promise for applications in patient rehabilitation, worker performance monitoring, and as a human-machine interface for controlling robots to operate hazardous tasks. Movie S5 (2× speed) records physiological signals and joint movements.

### Comprehensive assessment of wearable electrophysiology under various conditions

**Fig. 4.**
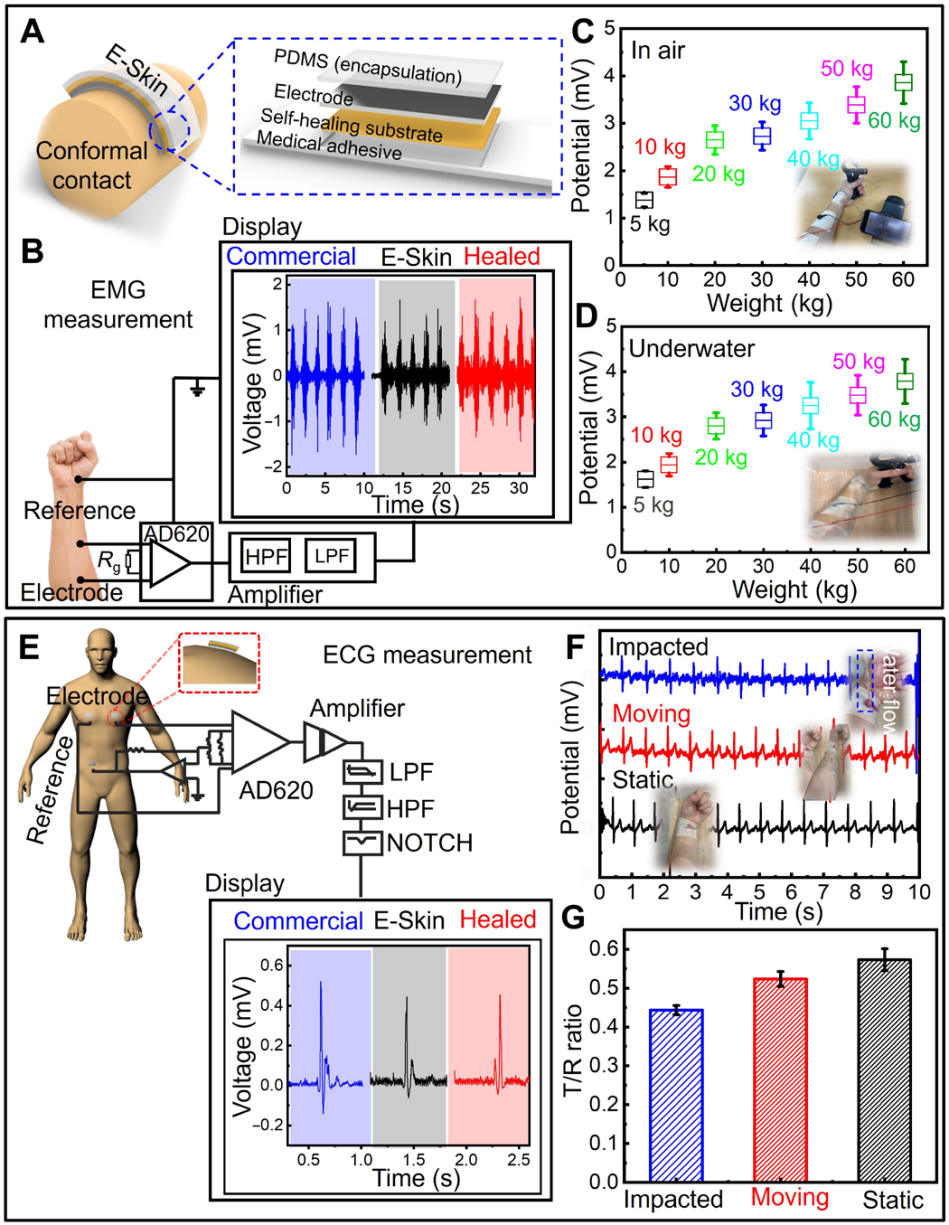
Comprehensive evaluation of wearable electrophysiological measurements using the E-Skin under diverse conditions. (**A**) Schematic illustrating the convenient and conformal attachment of the E-Skin to the epidermis, along with a magnified view of its 3D network structure. (**B**) Real-time comparison of sEMG measurements using a commercial sEMG Sensor, the E-Skin, and the healed E-Skin. (**C**) sEMG signals recorded by the E-Skin on the forearm under different grasping forces. (**D**) sEMG captured underwater with the E-Skin. (**E**) Real-time ECG measurements compared among a commercial ECG sensor, the E-Skin, and the healed E-Skin. (**F**) ECG signals recorded underwater, with corresponding photos illustrating different recording conditions: static, moving, and water-impacted. For the latter, water was directed through a 6.0-mm-diameter pipe at a flow rate of 2.0 liter/min. (**G**) Graphs detecting T/R peak ratio measurements corresponding to (F). LPF, low pass filter; HPF, high pass filter.

Wearable electrophysiological monitoring, particularly sEMG and ECG, is crucial for identifying potential safety risks and health issues in real-life scenarios, such as heart attacks or muscular injuries (*35*, *36*). For stable and reliable signal acquisition, the E-Skin was conformally attached to the epidermis (Fig. 4A). The E-Skin was placed on the forearm, and sEMG signals were recorded during muscle clenching and relaxing using a local field potential amplifier equipped with integrated high-pass and low-pass filters (Fig. 4B and movie S6). To improve signal quality and minimize noise, we used band-pass filters designed for the specific frequency ranges of the measurements: 10 to 500 Hz for EMG and 0.3 to 40 Hz for ECG. For enhanced signal-to-noise ratio (SNR), we used a three-electrode configuration instead of a two-electrode system, incorporating a third electrode connected to a drive-right-leg circuit to reduce power line noise. The collected data were then imported into MATLAB for further processing, where we applied a digital Butterworth filter and a notch filter with a sharper slope to effectively clean the data. A more detailed clarification of the numerical computation methods used for data processing and analysis is provided in note S7.

Furthermore, the developed E-Skin exhibited rapid stability in sEMG recording within 10 s post-healing under ambient conditions, achieving performance comparable to existing technologies (fig. S18). The sEMG signal processing workflow is detailed in fig. S19. When applied to the forearm and synchronized with a hand dynamometer, the E-Skin detected an increase in EMG signal intensity corresponding to increased grasping force (Fig. 4C). The E-Skin maintained its functionality underwater, effectively tracking muscle activity as its conductive and adhesive properties remained intact during water immersion (Fig. 4D, fig. S20, and movie S7).

Consistent with the sEMG measurements, ECG recordings were reliably achieved using the E-Skin device paired with a cost-effective AD620 instrumentation amplifier (Fig. 4E). The self-healing capability of the E-Skin quickly restored its functionality, yielding signal quality comparable to that of an intact E-Skin measured in static air conditions (fig. S21). To assess underwater ECG recording performance with the E-Skin, we tested three conditions: without arm motion (static), with arm motion (moving), and with water flow impacting the E-Skin (affected) (Fig. 4F, figs. S22 and S23, and movie S8). Given that most underwater environments involve dynamic water flow, the affected condition offers a more accurate representation of real-life applications. The recorded data were analyzed for quality and stability to enable a comprehensive comparison. The ECG signals were characterized by their key components: the P wave, the QRS complex, and the T wave (fig. S24) (*37*). The R wave appears during ventricular contraction, representing the heart’s pumping function, while the T wave (around a third of the size of the R wave) indicates the relaxing and blood-filling phase of the ventricles (*38*). Labor-intensive tasks increase ventricular relaxation, enlarging the T wave. As shown in Fig. 4G, the T/R ratios recorded during movement and water impact were similar to those recorded under static conditions due to the tight contact of the E-skin with the epidermis. We expanded our experimental scope to encompass a broad range of dynamic and complex conditions, aiming to better simulate real-world scenarios and further validate the device’s reliability and adaptability. As shown in fig. S25, ECG measurements were conducted under diverse aquatic conditions, including variations in water temperature (10°, 25°, and 40°C), depth (0, 30, and 60 cm), and type (sea water and tap water), as well as during dynamic movements such as swiveling, swaying, and flapping. These results demonstrate the device’s capability to maintain stable ECG measurements across varying underwater environments and during different dynamic motions.

To elucidate the potential variations in ECG signals across different age groups and health conditions, we conducted additional experiments to expand our dataset. Figure S26A presents EMG signals recorded from adolescents (ages 10 to 19), vicenarians (ages 20 to 29), and tricenarians (ages 30 to 39) following a high-intensity strength exercise (30 push-ups, along with grip strength measurements). The results indicate that younger individuals exhibit stronger EMG signals and faster muscle responses. Figure S26B demonstrates that younger participants tend to have higher heart rate variability and normal ECG waveforms. ECG signals recorded post a 100-m sprint show that participants across all age groups (adolescents, vicenarians, and tricenarians) experienced an increased heart rate following exercise. A detailed clarification of differences in ECG signals across different ages and health conditions is provided in note S8. Furthermore, we conducted on-skin tests to evaluate the long-term wearability of the E-Skin. For comparison, two patches (the E-skin and a commercial gel electrode) were applied to the forearms of an adult subject for 1 day. As shown in fig. S27, the E-skin caused no adverse skin reactions with daily use. In contrast, the commercial gel electrode resulted in noticeable skin erythema, likely due to its limited oxygen and moisture permeability.

### Machine learning–empowered prediction and classification of muscle fatigue

The time-domain and frequency-domain features of sEMG signals are well-established indicators for assessing muscle strength and fatigue (*39*–*43*). Key biometric parameters—such as the average rectified value (ARV), root mean square (RMS), mean frequency (MEF), and median frequency (MDF)—have been extensively used to quantify muscle fatigue. Time-domain metrics, including ARV and RMS, offer insights into muscle fatigue and are instrumental in estimating endurance time. Moreover, alternations in the frequency-domain patterns of sEMG are associated with reductions in muscle force from an unfatigued state (*44*, *45*). Our analysis of sEMG signals, as detailed in table S3, enabled us to quantify muscle fatigue and strength effectively.

We used a machine learning–powered system to predict and classify muscle fatigue by analyzing time- and frequency-domain features of sEMG signals obtained from the E-Skin. The system integrates wireless electronics with embedded algorithms, offering advanced neurorecording capabilities and comprehensive sEMG pattern recognition. The electronic circuit of the E-Skin system is composed of a bluetooth low energy (BLE) system-on-chip (SoC) system that is responsible for acquiring data from the analog front-ends (AFEs) dedicated to sEMG signals. The BLE SoC enables wireless data communication to a graphic user interface, which houses a compressed AI model, and a specialized deep-learning inference framework optimized for real-time wearable applications (figs. S28 and S29). These embedded components facilitate real-time sEMG signal pattern recognition and accurate prediction of muscle fatigue levels.

**Fig. 5.**
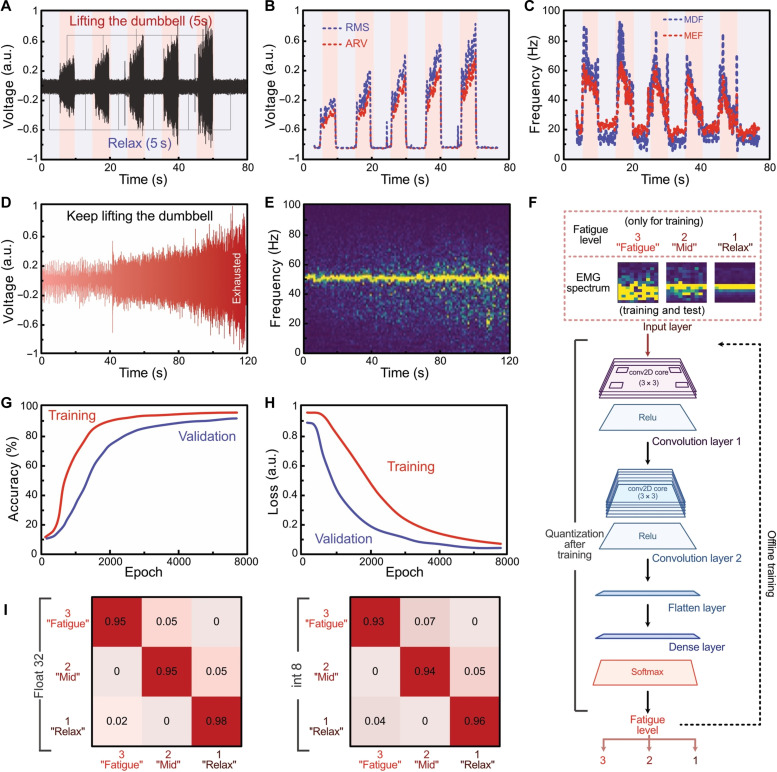
Machine learning–based muscle fatigue prediction and classification. (**A**) Raw sEMG signal data from the E-Skin during a sequence of lifting a dumbbell, holding the lifted position for 5 s, lowering it, and relaxing for another 5 s. (**B** and **C**) Analysis of the raw sEMG data in both the time domain (ARV and RMS) and the frequency domain (MDF and MEF). (**D**) Real-time sEMG signals at different levels of muscle fatigue, used as the training dataset for the machine learning model. (**E**) Time-frequency diagram of the sEMG signals generated under continuous muscle exertion for 2 min. (**F**). Structure of the machine learning model. (**G** and **H**) Changes in accuracy and loss changes during the training process. (**I**) True- and false-positive plots under various quantification conditions. a.u., arbitrary units.

We used this machine learning model to analyze sEMG signals for the assessment of muscle fatigue, with data gathered via the E-Skin. Figure 5 provides an overview of the methodology, detailing a series of analyses and model training processes. Raw sEMG signals were initially captured as subjects performed a standardized dumbbell exercise regimen. The study involved 21 subjects, with their characteristics detailed in table S4. The protocol consisted of a cyclic sequence where subjects lifted and held a dumbbell for 5 s, followed by a 5-s rest period. Additional details regarding the protocol are available in note S9 and table S5. This sequence was repeated to generate a robust dataset representing varying levels of muscle fatigue (Fig. 5A).

We then extracted temporal- and frequency-domain features from the data presented in Fig. 5A. The time-domain analysis focused on computing ARV and RMS to indicate signal magnitude and muscular activity. Concurrently, the frequency-domain analysis used MDF and MEF to characterize muscle fatigue over time (fig. S30). As muscle fatigue increased, initial increments in the RMS and ARV values were observed due to the recruitment of additional muscle fibers. However, as fatigue intensified, a reduction in muscle fiber capability led to decreased RMS values. Both MDF and MEF generally declined with increasing muscle fatigue, indicating a shift in the signal toward a lower frequency as fatigue progressed. As shown in Fig. 5 (B and C), ARV and RMS values increased, while MDF and MEF values decreased with an increase in fatigue induced by repeated lifting of a 5-kg dumbbell. To provide a more comprehensive analysis, the subjects continued the dumbbell lifting exercise for 2 min post-rest, during which sEMG signals were captured to monitor the onset and progression of muscle fatigue. This extensive dataset provided a suitable training dataset for the machine learning algorithm (Fig. 5D).

To investigate the interplay between temporal evolution and spectral changes in muscle activity, we visualized the time-frequency distribution of the sEMG signal, providing insights into spectral patterns associated with sustained effort (Fig. 5E). For this purpose, we developed a two-layer convolutional neural network (CNN) model to classify levels of muscle fatigue from the sEMG data (Fig. 5F). The model’s architecture includes an initial input layer, followed by a first 2D convolution layer with rectified linear unit (ReLU) activation to enhance computational efficiency, and a second 2D convolution layer to further extract fatigue-related features, both using 8 × 10 filters. The output from the second convolution layer is passed through an additional ReLU activation layer, flattened, and fed into a fully connected layer for feature mapping, ultimately producing the final fatigue score via SoftMax activation.

We divided the precollected EMG data into three sets to train the model: 60% for training, 20% for validation, and 20% for testing. The CNN model was trained while minimizing the cross-entropy loss function, with a dropout rate of 0.5 applied to mitigate overfitting. After 100,000 epochs, the CNN model achieved an accuracy exceeding 90% (Fig. 5, G and H). Subsequently, the model parameter values were fixed, and quantization was applied, resulting in an 8-bit model with a reduced size of less than 20 kB, facilitating integration with the real-time graphic user interface (movie S9).

We assessed the classification performance of the CNN model using confusion matrices to quantitatively represent the model’s predictions across three distinct classes: Fatigue, medium (Mid), and relax. As shown in Fig. 5I, the matrix on the left depicts the performance using 32-bit floating-point precision, while the matrix on the right illustrates the performance after applying 8-bit integer quantization. In these confusion matrices, the diagonal elements indicate the proportion of correct predictions for each class, while the off-diagonal elements represent misclassifications.

The 32-bit model demonstrated high accuracy in classifying different states, achieving 96% for “Fatigue,” 95% for “Mid,” and 98% for “Relax.” Similarly, the quantized 8-bit model showed strong performance, with classification accuracies of 93% for Fatigue, 94% for Mid, and 96% for Relax. These results indicate that the model preserves high predictive accuracy following quantization. While quantization typically reduces the memory footprint and computational complexity of the model at the cost of some accuracy, our CNN model maintains high-performance post-quantization. This underscores its potential for real-time fatigue monitoring in wearable devices.

## DISCUSSION

We developed a rapid self-healing E-Skin patch for noninvasive real-time electrophysiological and joint-movement monitoring. The E-Skin was constructed from stretchable PTMEG-modified TPU and a soft silver nanowire network that enabled direct and intimate contact with the skin. This design ensured self-healing within 10 s, without the need of external stimuli, and under ambient conditions, effectively responding to mechanical damage resulting from the skin’s dynamic movements, providing adaptability suitable for real daily life use. This was achieved by integrating bis(4-hydroxyphenyl) disulfide into the TPU matrix, which endowed the E-Skin with self-healing capabilities through the formation of intracellular disulfide bonds. IPDI, characterized by its an asymmetric alicyclic structure, was used to enhance chain mobility, thereby facilitating disulfide exchange. This modification further improved material’s self-healing capabilities across a broad temperature range, including both high temperature and low temperatures. This significantly mitigated mechanical damage from repeated wear, tear, and accidental cuts or scratches, thereby preserving the device’s structural integrity and functionality over prolonged use.

Integrating E-Skin with wireless electronics and embedded algorithms enabled the long-term monitoring of biophysical signals and facilitated on-site AI-powered analysis. This system provided real-time identification and classification of muscle fatigue. Leveraging a CNN algorithm to learn from sEMG signal patterns associated with fatigue, the system offers a robust tool to monitor the muscular status of the users and potentially supply an early warning of injury risk due to muscle fatigue. The seamless incorporation of AI and embedded algorithms further enhanced its utility in health evaluation and rehabilitative therapies.

Despite the significant performance of the E-Skin device, future advancements focus on several key areas: First, enhancing electrode materials and mechanical structure is essential; while AgNW has shown effectiveness, alternative materials such as MXene, CNTs, and conductive polymers hold additional potential. Future research will prioritize exploring these materials individually and in combination to optimize device performance, aiming for improved stability, reliability, and cost-effectiveness. Second, extending the operational duration of the E-Skin is crucial to facilitate long-term electrophysiological monitoring in real-life scenarios, with a goal of enabling the device to function continuously for days or even weeks. Third, achieving full miniaturization of the E-Skin will require the development of integrated electronics capable of supporting electro-impedance spectroscopy, signal processing, and wireless transmission. By creating a standalone E-Skin sensing interface equipped with AI-assisted on-site signal processing, the device can be transformed into a comprehensive, skin-interfaced monitoring system. Last, conducting large-scale validation studies across diverse populations is essential, given the variability of physiological signals. These studies will ensure the E-Skin functions reliably for a wide range of users, paving the way for a fully integrated, intelligent platform that provides valuable insights into individual health and physiological status, contributing to injury prevention and management, particularly in labor-intensive occupations.

We envision this rapidly self-healing E-Skin patch offers substantial benefits to a diverse group of users—including construction workers, athletes, and military personnel—by enabling robust health monitoring in daily life and under extreme conditions. It aims to prevent and manage injuries related to labor-intensive activities, optimize training regimens, and support physical therapy by real-time tracking recovery progress. Ultimately, this E-Skin patch has the potential to enhance overall health and performance across various real-world scenarios.

## MATERIALS AND METHODS

### Materials

PTMEG with an average molecular weight of 1000 g mol^−1^, IPDI (98%, mixture of isomers), and dibutyltin dilaurate were obtained from Sigma-Aldrich. Dimethylacetamide was sourced from Samchun Chemical, and bis(4-hydroxyphenyl) disulfide was acquired from Ambeed. All reagents were used as received without further purification.

### Self-healing polymer fabrication

PTMEG (15 g) was dried at 100°C for 1 hour to remove residual moisture and then cooled to 70°C. Meanwhile, IPDI (10 g, 44.98 mmol) and dibutyltin dilaurate (0.2 g) were dissolved in 5 ml of *N*,*N*-dimethylacetamide (DMAc) under a nitrogen (N_2_) atmosphere for 2 hours and then cooled to room temperature. Bis(hydroxyphenyl) disulfide (3.63 g, 14.50 mmol), dissolved in 10 ml of DMAc, was introduced into the reactor as a chain extender. The reactor temperature was raised to 40°C, allowing the reaction to continue until the NCO peak was no longer detectable. The concentration of the resultant polymer solution was adjusted to 30 weight % by adding 43 ml of DMAc. The solution was then degassed under vacuum for approximately 1 hour to eliminate bubbles. Subsequently, 20 ml of the synthesized polymer solution was poured onto a Teflon-coated dish, cured on a hotplate at 40°C for 1 day, and then allowed to cure at room temperature.

### E-Skin fabrication

The E-Skin substrate was treated with utraviolet (UV)–Ozone (UVC-30s, Jasung Engineering) to eliminate potential organic contaminants and introduce oxygen-containing functional groups, thereby enhancing its hydrophilic properties. Silver nanowire ink was prepared following our established methods (*23*, *28*). The silver nanowire electrodes were precisely patterned onto the self-healing E-Skin substrate using screen-printing techniques facilitated by laser-cut shadow masks. The printed E-Skin was then annealed to improve its stability at room temperature.

### On-body validation of the E-Skin

The epidermal evaluation was conducted on healthy adult individuals without heart conditions, diabetes, or chronic pain, adhering strictly to the protocol sanctioned by the Institutional Review Board at the University of California, Los Angeles (IRB#17-000170). Further, informed written consent was obtained from all participants.

### ECG measurement

The E-Skin was applied to the forearm for ECG measurements. Two E-Skin patches were positioned on the volunteer’s left and right forearms secured with a commercially available medical adhesive (3M, USA) to ensure proper adhesion. ECG signals were acquired using a commercial ECG board (SparkFun AD8232 ECG monitor) and recorded with a digital phosphor oscilloscope (Tektronix, DPO3014). The electronic circuit board was powered by a portable power source (Dr. Meter, PS-305DM) at approximately 3.3 V. The circuit board utilized an AD8232 chip to amplify and filter the raw ECG signals.

### Electrical integration for sEMG recording

We incorporated a BLE SoC (NRF52840-CKAA, Nordic Semiconductor) encased in a compact wafer-level chip-scale package (WLCSP) measuring 3.5 mm by 3.6 mm, serving as both the central processing unit and Bluetooth communication module to facilitate deep-learning operations. A rechargeable lithium-polymer battery, connected to a low-dropout linear voltage regulator (ADP7112, Analog Devices), provided a stable voltage output. For wireless data transmission, the BLE SoC used a miniature ceramic antenna (2450AT18A100, Johanson Technology) measuring 3.2 mm by 1.6 mm and operating at 2.45 GHz. The amplification, filtering, and digitization of sEMG signals were performed using ultralow-power biopotential AFEs (MAX30003, Maxim Integrated) in a WLCSP format measuring 2.74 mm by 2.9 mm. These AFEs exhibited exceptional performance, with an ultralow-power consumption of 180 μW and a high common-mode rejection ratio exceeding 100 dB. Digital sEMG data were transmitted to the BLE SoC via the serial peripheral interface. To ensure a stable 1.8-V supply for the biopotential AFE, a secondary low-dropout voltage regulator (LP5907UVX, Texas Instruments) in a four-pin WLCSP measuring 0.645 mm by 0.645 mm was connected to the output of the primary voltage regulator. The integration of passive components in 0201 packages, known for their compact size, helped minimize the system’s overall physical footprint.

### Characterizations and measurements

The transmittance spectra of PDMS and E-Skin were recorded using a Shimadzu UV-visible spectrophotometer. The strain-stress response of the E-Skin during the healing process was analyzed with a JSH-H1000 Horizontal Acomatic Handy Test Stand and SOP-EG1 software. The applied force was quantified using a Mark-10 force monitor, and the corresponding output characteristics were captured with an MDO-3052 oscilloscope. This oscilloscope was also used to record output characteristics from various body locations. The healing process of the E-Skin following complete severance was assessed using AFM (NX10). For EMG and ECG measurements, a PZ5 neuro digitizer amplifier and RZ2 software were used.
