# Supplementary material for: Rapidly self-healing electronic skin for machine learning–assisted physiological and movement evaluation
Source: Sci Adv. 2025 Feb 12;11(7):eads1301. doi: 10.1126/sciadv.ads1301 (PMC11818020; doi:10.1126/sciadv.ads1301)
Supplement: Supplementary file 1 — Supplementary Text Figs. S1 to S30 Tables S1 to S5 Legends for movies S1 to S9 [file sciadv.ads1301_sm.pdf]

Science Advances
AAAS

Supplementary Materials for

Rapidly self-healing electronic skin for machine learning-assisted physiological and movement evaluation

Yongju Lee *et al.*

Corresponding author: Hyeok Kim, hyeok.kim@uos.ac.kr; Ali Khademhosseini, khademh@terasaki.org; Yangzhi Zhu, yzhu@terasaki.org

Sci. Adv. **11**, eads1301 (2025)
DOI: 10.1126/sciadv.ads1301

The PDF file includes:

Supplementary Text
Figs. S1 to S30
Tables S1 to S5
Legends for movies S1 to S9

Other Supplementary Material for this manuscript includes the following:

Movies S1 to S9

Supplementary Text

Supplementary Note 1. Clarification of the biocompatibility of the IPDI-based TPU

Isophorone diisocyanate (IPDI) in its raw state is highly reactive and toxic, presenting considerable challenges. However, through controlled polymerization, IPDI-based polyurethanes are synthesized by a complete reaction between IPDI and polyols, yielding stable urethane linkages that neutralize the reactive isocyanate groups. This transformation alters IPDI's chemical profile; in our properly synthesized polyurethane, all IPDI is fully reacted, with no residual unreacted IPDI remaining, thereby eliminating the toxicity associated with the raw monomer.

The resulting IPDI-based polyurethane matrix is chemically distinct from IPDI itself, exhibiting stability and inertness in biological settings. Unlike raw IPDI, which is volatile and highly reactive, the polymerized polyurethane is solid, non-reactive, and resistant to hydrolysis, reducing the likelihood of adverse biological responses upon tissue contact.

In this work, the fabrication process for IPDI-based polyurethanes includes critical steps to remove any remaining monomers or reactive groups, utilizing post-processing techniques such as curing and washing to ensure the final material is free from any potentially harmful residues. Therefore, the biocompatibility of IPDI-based polyurethanes arises not from the properties of IPDI itself, but from the stable, inert structure achieved through complete polymerization within the polyurethane matrix. This transformation from a reactive monomer to a stable polymer underscores a core principle in the design of biocompatible materials, offering a safe and reliable solution for diverse biomedical applications.

In addition, empirical evidence from extensive clinical use supports the biocompatibility of IPDI-based polyurethanes, further reinforced by rigorous testing in alignment with standards like ISO 10993. Biocompatibility assessments, including cytotoxicity, hemocompatibility, and sensitization testing, confirm that IPDI-based polyurethanes meet the stringent demands of biomedical applications, even for long-term on-body use *(18.19)*.

Supplementary Note 2. Clarification of the self-healing mechanism of the E-Skin

The self-healing mechanism of our E-Skin is fundamentally based on the formation of reversible chemical bonds, particularly dynamic covalent bonds via Diels-Alder reactions. These bonds enable the material to autonomously repair itself at room temperature, eliminating the need for external intervention. The inherent flexibility of the polymer network is critical, as it allows the material to realign and restore its original structure following mechanical damage.

To further enhance this self-healing capability, isophorone diisocyanate (IPDI) was incorporated into the TPU matrix. The asymmetric alicyclic structure of IPDI increases the flexibility and mobility of the polymer chains, which is essential for effective self-repair. IPDI promotes the disulfide metathesis reaction within the TPU matrix, enabling reversible disulfide bonds to autonomously break and reform. This dynamic bonding process underpins the self-healing mechanism, allowing the material to repair itself without the need for heat or other external stimuli.

Our E-Skin stands out from existing self-healing materials due to its ability to heal at room temperature within 10 seconds, while many other systems require high heat, UV light, or other external triggers. Furthermore, our system provides the flexibility to accelerate the self-healing process at intermediate temperatures (60-90°C), enhancing repair efficiency without compromising the material’s mechanical integrity. This dual-mode healing, automatic at room temperature, with the option for faster repair at higher temperatures, ensures the polymer retains its original physical properties while delivering robust and efficient self-repair.

Supplementary Note 3. Rationale for the design of the E-Skin

To enhance the self-healing efficiency of the polymer, we carefully adjusted its molecular weight and crosslinking density to improve molecular mobility, thereby facilitating the rearrangement and bonding of molecules. By modifying the molecular weight of the soft segment, poly(tetramethylene ether glycol) (PTMEG), we effectively modulated the elasticity, toughness, and self-healing properties of the film. We examined PTMEG molecular weights of 200, 1000, and 20,000, each maintained at a fixed quantity of 15 g. Notably, PTMEG with a molecular weight of 20,000 did not dissolve in N,N-dimethylacetamide (DMAc), resulting in self-aggregation and solidification. This behavior impeded its interaction with other components, such as dibutyltin dilaurate (DBTDL) and isophorone diisocyanate (IPDI), thereby obstructing the formation of a self-healing matrix.

In contrast, films produced with PTMEG of molecular weight 200 exhibited a Young's modulus of approximately 10 kPa, significantly below the typical skin stiffness range of 0.1 MPa to 2 MPa. This lower modulus indicated insufficient strength and stability, making it unsuitable for E-skin sensor applications. Consequently, PTMEG with a molecular weight of 1000 was selected for its balanced properties, achieving an optimal combination of elasticity, toughness, and self-healing capability. This optimized formulation of PTMEG with a molecular weight of 1000 was employed in subsequent on-body validation experiments, forming the foundation for our E-Skin development.

Supplementary Note 4. Rationale for using AgNWs as electrode material in this study

Silver nanowires (AgNWs) are extensively utilized as flexible electrodes in wearable technologies, including E-Skin, due to their low electrical resistance, mechanical robustness, and flexibility. The one-dimensional nanowire network ensures stable electrical performance even under mechanical strain. Among metal nanowires, AgNWs stand out for their exceptional electrical and thermal conductivity, which is critical for ensuring the reliability and stability of E-Skin in practical applications.

While alternative electrode materials can enhance the versatility of E-Skin technology, a significant trade-off exists between achieving high conductivity and maintaining self-healing capabilities. In our study, we explored the use of carbon nanotubes (CNTs) within the self-healing polymer to fabricate the E-Skin device. As shown in Supplementary Figure 5, increasing the CNT content from 0.1 wt% to 1.5 wt% did not yield the desired conductivity, and further increases beyond 1.5 wt% resulted in a noticeable decline in self-healing properties. Consequently, CNTs may not be suitable for this application.

To address this limitation, we developed a self-healing polymer that incorporates AgNWs, which provide both high conductivity and flexibility. The integration of AgNWs enabled the self-healing polymer to retain the necessary conductivity for bio-signal detection while maintaining excellent mechanical flexibility, allowing the device to perform reliably under various deformation conditions. Additionally, the AgNW network structure facilitated rapid restoration of conductive pathways after damage, thereby enhancing the device’s durability and overall performance. To ensure optimal conductivity, the AgNW conductive patterns were printed onto the substrate.

Supplementary Note 5. Experimental conditions for evaluating self-healing performance

The experimental conditions in our study were meticulously selected to replicate real-world environments where the E-Skin device is likely to be deployed. Specifically, we evaluated the self-healing performance under humidity levels of 40%, 60%, and 80%, representing a spectrum of indoor and outdoor conditions, from relatively dry to highly humid settings. Temperature settings of 25°C, 40°C, and 60°C were chosen to mimic typical ambient temperatures encountered across various operational scenarios. In addition, we conducted tests in water to simulate conditions where the E-Skin might be exposed to moisture or submerged, reflecting common real-life situations. To enhance the practical relevance of our findings, we performed repeated self-healing tests under these conditions. The results demonstrated consistent performance without degradation, even after multiple cycles, confirming the E-Skin's reliability and robustness in these simulated environments.

Supplementary Note 6. Self-powered sensing mechanism of the E-Skin

The use of the triboelectric mechanism in E-Skin development is driven by its exceptional sensitivity to mechanical disturbances, which is crucial for the precise detection and response to various physical interactions. Triboelectric sensors can produce significant electrical outputs from minor deformations, making them ideal for applications requiring accurate touch and pressure detection. Furthermore, these materials are inherently flexible and can be seamlessly integrated into various substrates, ensuring that the E-Skin remains lightweight, flexible, and comfortable. This versatility is essential for applications in wearable technology, robotics, and healthcare, where the integration of comfort and functionality is paramount.

The functionality of E-Skin relies on the triboelectric effect, which operates through a cyclical process involving four distinct phases: the Pressed mode, where two triboelectric materials are in close proximity, facilitating maximal surface charge exchange; the Releasing mode, during which the materials separate, generating an electric field that propels current through an external circuit; the Released mode, characterized by maximum separation and a temporary cessation of electrical output as the surface charge stabilizes; and the Pressing mode, wherein the materials converge, reversing the electric field and charge flow, thus priming the system for the next cycle of energy conversion.

Supplementary Note 7. Data processing and analysis methods for E-Skin measurements

To ensure the accuracy and reliability of our results, we employed the following comprehensive approach:

1). Data Filtering: To mitigate noise in the collected data, we applied a low-pass filter, which effectively removed high-frequency noise from the sensor signals. This step was essential for isolating relevant data from the E-Skin, allowing for more precise analysis.

2). Signal Averaging: To reduce random errors and enhance the signal-to-noise ratio, we performed signal averaging across multiple trials. This technique improved the reproducibility and reliability of the data, ensuring consistent results.

3). Outlier Detection and Removal: We used an outlier detection algorithm based on statistical analysis, such as Z-scores, to identify and exclude any data points that significantly deviated from the expected range. This process ensured the integrity of the data set by removing anomalies that could skew the results.

4). Calibration: Before data collection, the E-Skin sensors were calibrated using a reference material with known properties. This calibration process was critical in adjusting the sensor outputs, ensuring that the collected data were accurate and fell within the expected range.

5). Validation with Control Experiments: To confirm the accuracy of our findings, we conducted control experiments. These control tests provided a benchmark for comparison with the E-Skin data, further validating the reliability and accuracy of our results.

These steps collectively ensured that the data from the E-Skin were processed with high precision, reducing noise and errors while maintaining the integrity and accuracy of the final results.

Supplementary Note 8. External Clarifications of differences in ECG signals across different ages and health conditions

ECG signals vary significantly with age and health conditions, reflecting changes in heart function and structure over time. Below is a detailed clarification of these differences based on age and health status.

1. Age-related Differences in ECG Signals

Heart rate variability (HRV): Younger individuals typically exhibit higher HRV, indicating a more adaptable autonomic nervous system. In older adults, HRV generally decreases, reflecting diminished autonomic flexibility and potential cardiac function impairment.

P-wave changes: In younger individuals, P-waves, which represent atrial depolarization, are generally of normal duration and amplitude. With age, P-waves may become wider or notched due to atrial enlargement or delayed conduction from structural heart changes.

PR interval: In younger individuals, the PR interval (time between atrial and ventricular activation) is typically normal. However, in older adults, this interval may lengthen due to slower electrical conduction through the AV node, a common aspect of aging.

QRS complex: In younger individuals, the QRS complex (ventricular depolarization) is usually narrow and normal in amplitude. In older adults, it may widen, often due to left ventricular hypertrophy, increased conduction time, or structural changes in the heart.

ST segment and T-wave: Younger individuals often have normal ST segments and T-waves, indicating healthy ventricular repolarization. In older adults, ST-segment depression or T-wave flattening/inversion is more frequent, possibly indicating ischemia or age-related heart conditions.

QT interval: The QT interval (time for ventricular depolarization and repolarization) is usually shorter in younger individuals. In older adults, a prolonged QT interval is more common, increasing the risk of arrhythmias and sudden cardiac death.

2. Health Condition-Related Differences in ECG Signals

Hypertension: Prolonged hypertension often leads to left ventricular hypertrophy (LVH), which is seen as an increased QRS amplitude and sometimes a widened QRS complex. ST depression and T-wave inversion may also occur due to cardiac strain.

Coronary artery disease (CAD)/ischemia: ECG changes such as ST-segment depression or elevation, and T-wave inversions, are common indicators of ischemia. In cases of previous myocardial infarction, pathological Q-waves may be present, indicating permanent damage.

Arrhythmias: Atrial Fibrillation (AF), characterized by the absence of distinct P-waves and irregular R-R intervals, are more common in older individuals and those with cardiovascular diseases, while Ventricular Tachycardia (VT), presenting with wide, rapid QRS complexes, is often observed in individuals with structural heart disease or previous myocardial infarctions.

Heart failure: Typical ECG signs include QRS prolongation, left bundle branch block (LBBB), and non-specific ST and T-wave changes, reflecting impaired electrical conduction and weakened cardiac muscle.

Diabetes: Diabetic patients are more prone to silent myocardial ischemia, often seen as non-specific ST-segment changes. Prolonged QT intervals are also common, increasing the risk of arrhythmias.

Obesity: Obesity can result in increased QRS amplitude and a rightward axis deviation. Increased body mass may also cause sinus tachycardia and left atrial enlargement, manifesting as P-wave changes.

Pulmonary conditions (*e.g.*, Chronic Obstructive Pulmonary Disease - COPD): COPD can cause right ventricular hypertrophy and right atrial enlargement, leading to an ECG pattern known as "P pulmonale" (tall, peaked P-waves in the inferior leads), as well as right axis deviation and low voltage QRS complexes.

Electrolyte imbalances: For hyperkalemia, high potassium levels can cause peaked T-waves, a widened QRS complex, and eventual cardiac arrest if untreated. For hypokalemia, low potassium levels can lead to flattened T-waves, the appearance of U-waves, and prolonged QT intervals.

Congenital heart conditions: Individuals with congenital defects such as ventricular septal defects (VSD) or atrial septal defects (ASD) may exhibit ECG abnormalities, including axis deviations, ventricular hypertrophy, or arrhythmias in severe cases.

Supplementary Note 9. Protocol to generate muscle fatigue

Task: Subjects were asked to use their bicep muscle (shoulder) to lift a dumbbell that weighed 60% of their maximum bicep (shoulder) muscle strength：

I. Subjects were asked to subject were asked to maintain a constant posture (90 degree of angle) to minimize variations in different tasks.

II. EMG electrodes were placed parallel with the muscle fiber between the motor point and the tendinous insertion, near the center of the muscle.

III. The Borg Rate of Perceived Exertion scale was used as a baseline to assess subjects' perceived exertion.

IV. Subjects were asked to rate their upper body muscles (shoulder muscle and bicep muscle) fatigue level every 15s.

V. According to subjects' perceived exertion, the recorded EMG signals were divided into: Relax or Low Fatigue Level (RPE scale between 0 and 2), Medium Fatigue Level (RPE scale between 3 and 6), High Fatigue Level (RPE scale between 7 - 10).

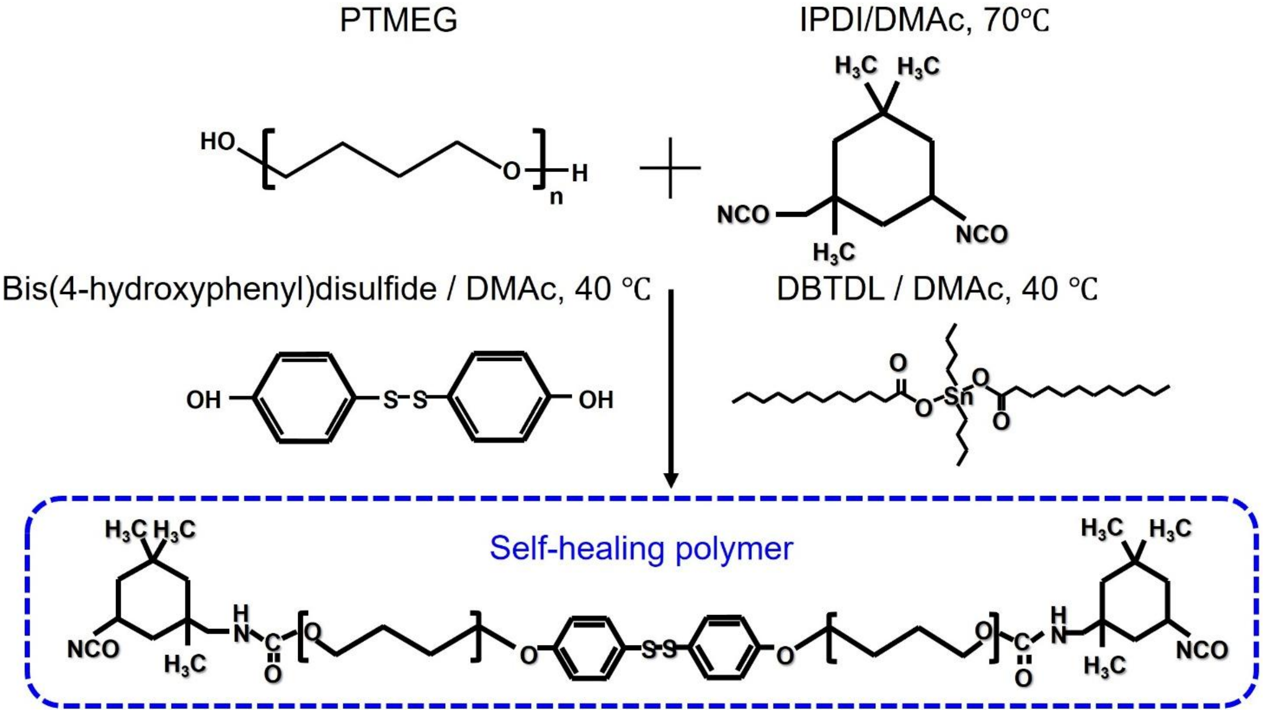

Fig. S1.

Molecular design of the E-Skin with self-healing capabilities. The process involves several critical steps: initially, polytetramethylene ether glycol (PTMEG) is prepared as the primary polymer backbone. Next, PTMEG reacts with isophorone diisocyanate (IPDI) and a dibutyltin dilaurate (DBTDL) catalyst under a nitrogen atmosphere to prevent oxidation and side reactions. The polymer then undergoes a chain extension process with bis(hydroxyphenyl) disulfide, which imparts self-healing properties to the material. Finally, the concentration of the polymer solution is adjusted to optimize the self-healing performance.

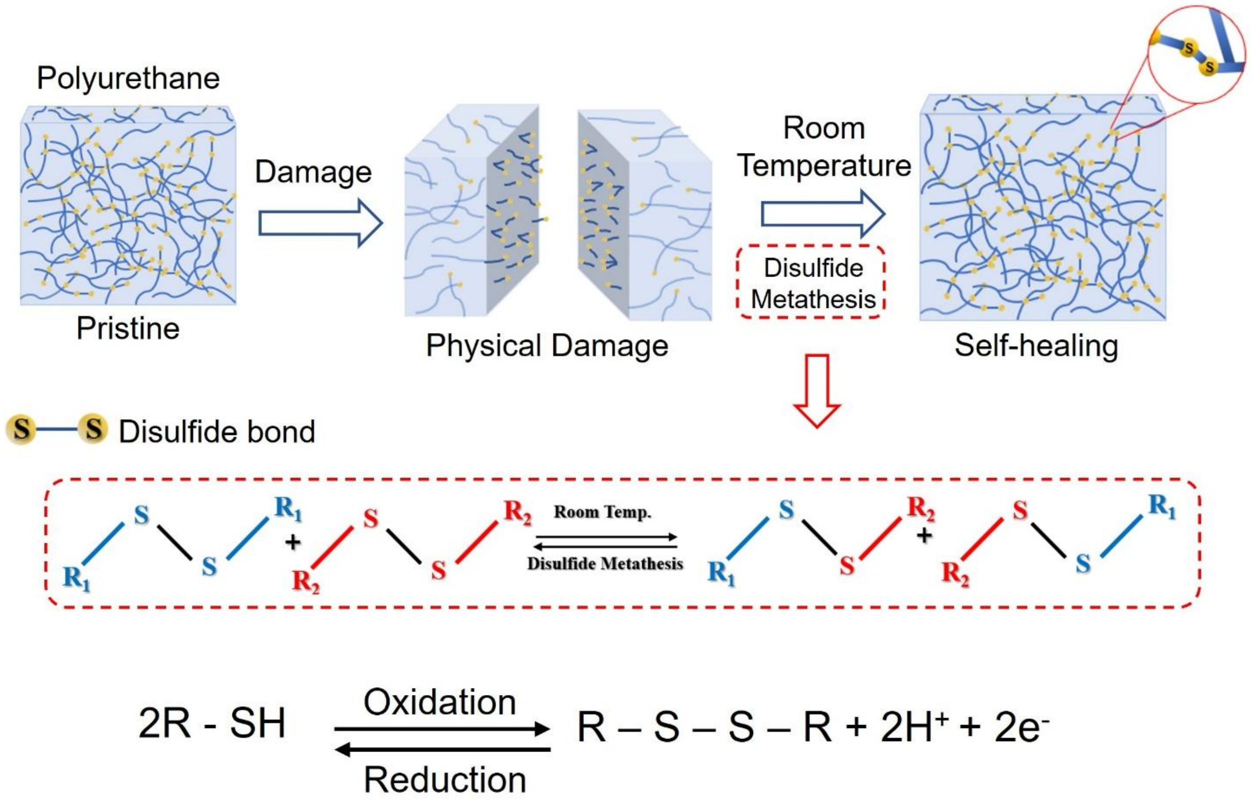

Fig. S2.

Mechanism revelation of the self-healing E-Skin. The self-healing ability is primarily governed by disulfide metathesis. This figure illustrates the detailed chemical reactions involved in the formation and dissociation of disulfide bonds throughout the healing process.

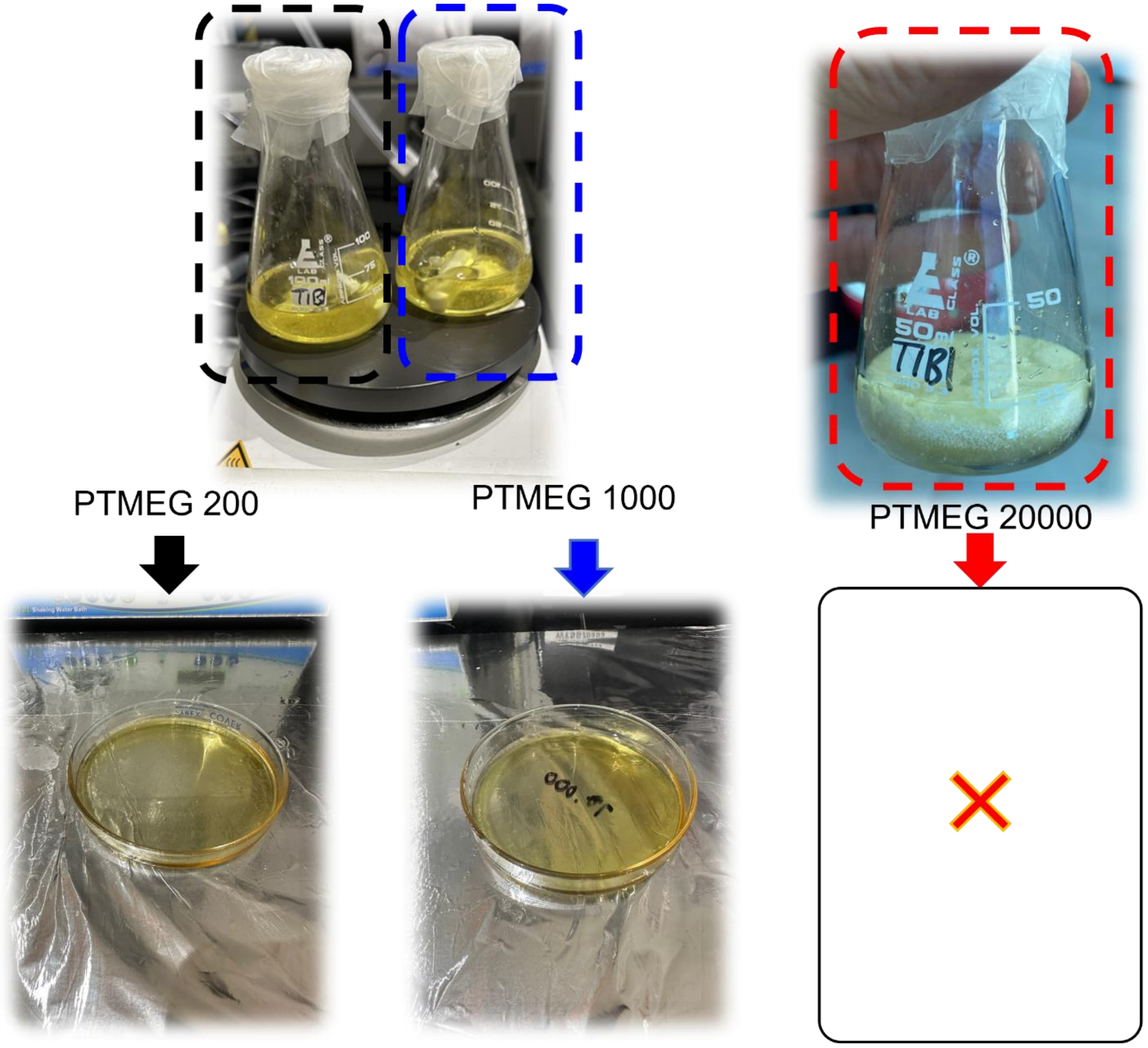

Fig. S3.

Influence of polytetramethylene ether glycol (PTMEG) molecular weights (Mw) on the mechanical properties of E-Skin. Evaluation of PTMEG at Mw 200, Mw 1000, and Mw 2000 reveals that the optimal molecular weight (PTMEG Mw 200) achieves a balance between strength, flexibility, and self-healing efficiency, enhancing E-Skin's suitability for intended applications.

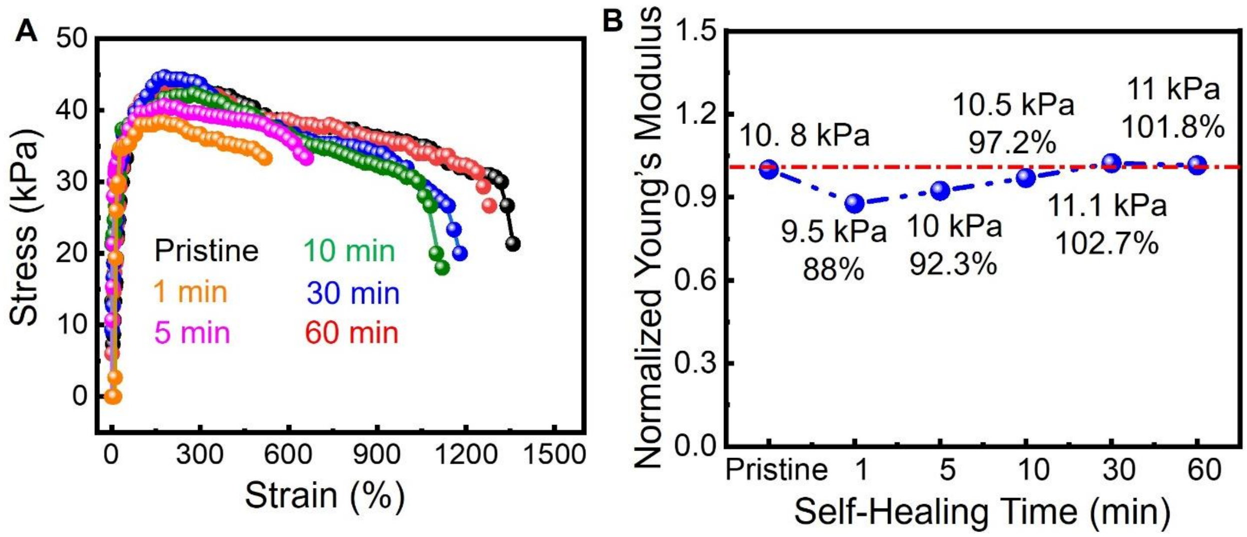

Fig. S4.

Self-healing performance of the E-Skin based on PTMEG (Mw 200). (**A**) Stress-strain curves demonstrating the mechanical property recovery of the E-Skin at various healing intervals (1, 5, 10, 30, and 60 minutes) post complete severance. (**B**) Young's modulus profile over different healing times, collected from the E-Skin synthesized with PTMEG of molecular weight 200.

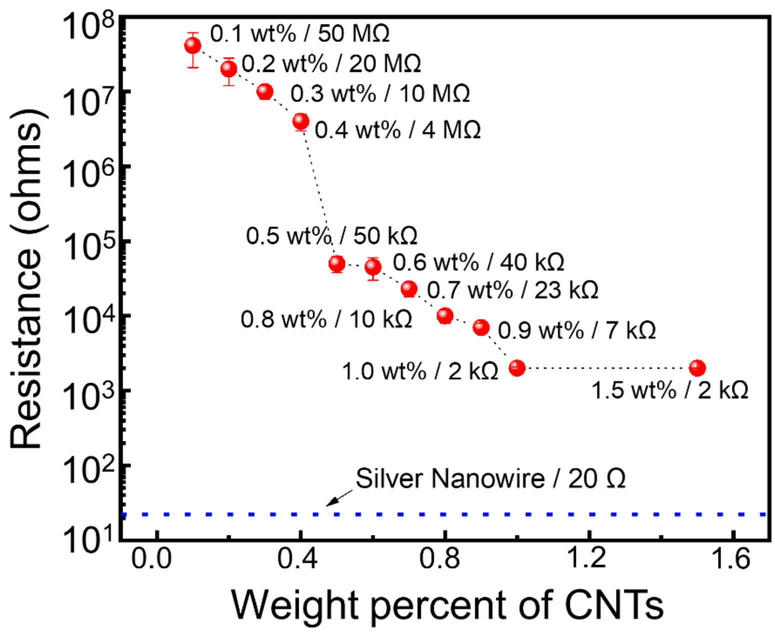

Fig. S5.

Conductivity of self-healing polymers as a function of CNT content. Raising the CNT content from 0.1 wt% to 1.5 wt% failed to achieve the desired conductivity, and further increases beyond 1.5 wt% led to a marked reduction in self-healing capabilities.

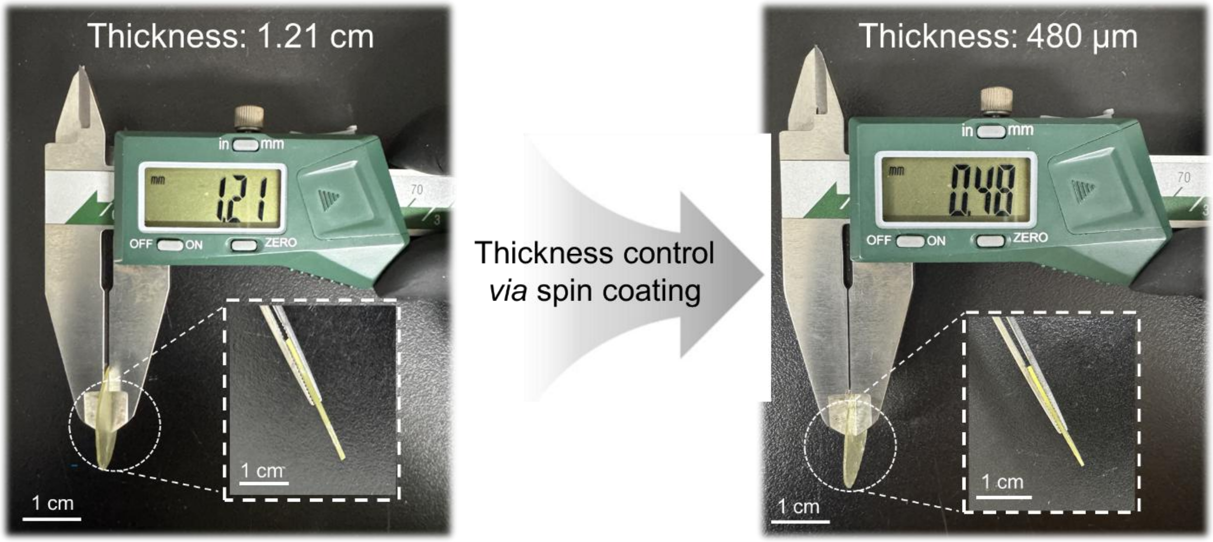

Fig. S6.

Thickness control of the E-Skin patch. The polymer solution is deposited onto a superhydrophobic-treated Si wafer and spin-coated at 150 rpm for 30 seconds, followed by curing. This modification has enabled us to achieve a device thickness of 480 μm, representing a significant reduction of approximately 60%.

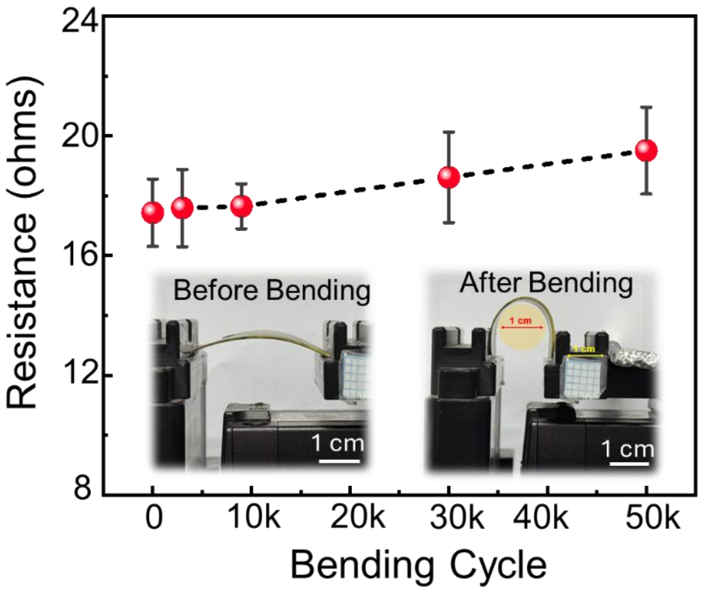

Fig. S7.

Mechanical stability of the E-Skin under repeated bending tests. Minimal conductivity variation was observed during the bending tests with a 0.5 cm bending radius, even after 50000 cycles.

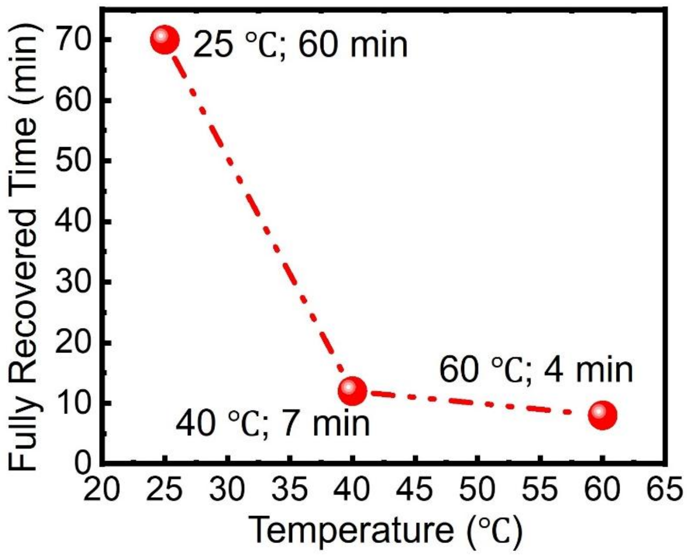

Fig. S8.

Impact of temperature on the self-healing performance of the E-Skin. This trend suggests temperature-dependent behavior in the self-healing mechanism of the E-Skin.

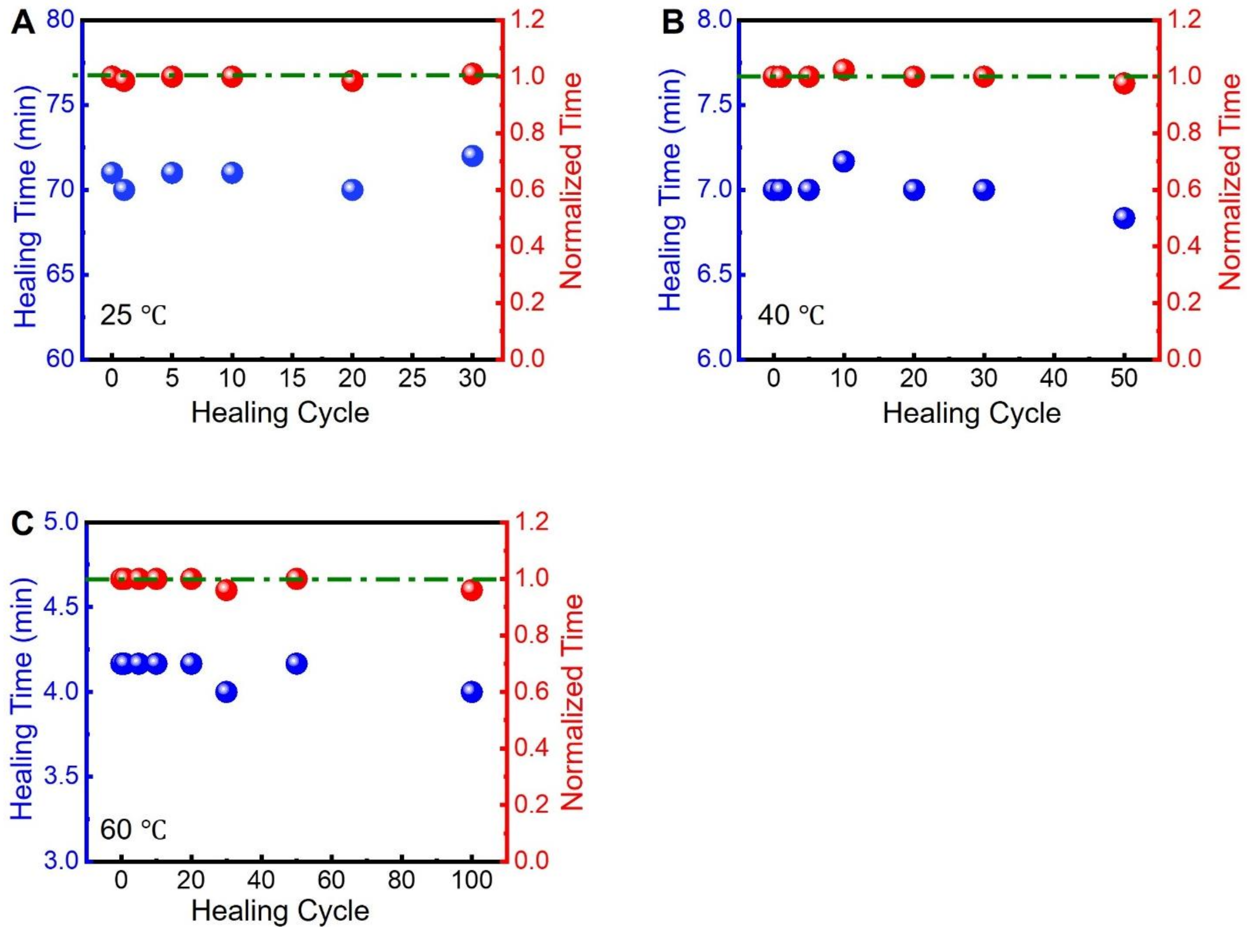

Fig. S9.

Performance and repeatability of the E-Skin across various temperatures: 25℃ (A), 40℃ (B), and 60℃ (C). The results demonstrate that elevated temperatures accelerate the self-healing performance of the E-Skin, and that this self-healing performance is consistent and repeatable under each specific condition. The blue data points represent the time required for the E-Skin to self-heal at each temperature, while the red data points indicate the normalized healing efficiency relative to a standard reference (dashed green line).

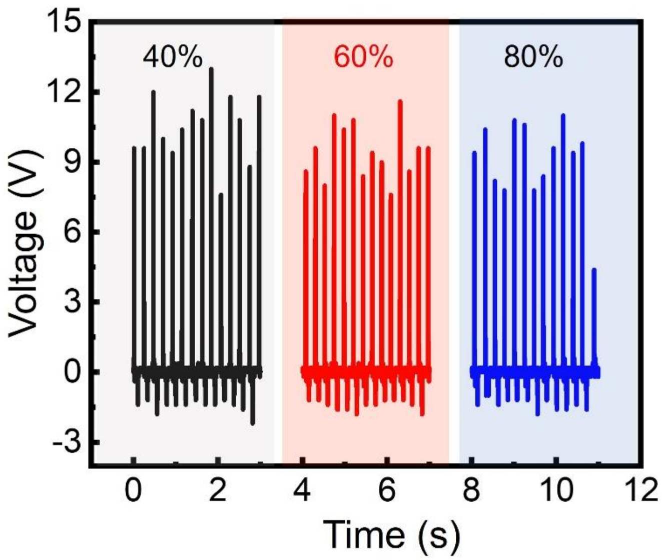

Fig. S10.

Pressure sensing performance of E-Skin across varying humidity levels (40%, 60%, 80%). Comparison of the E-Skin's pressure sensing capability under a constant force of 0.2 N, evaluated across different humidity conditions (40%, 60%, 80%).

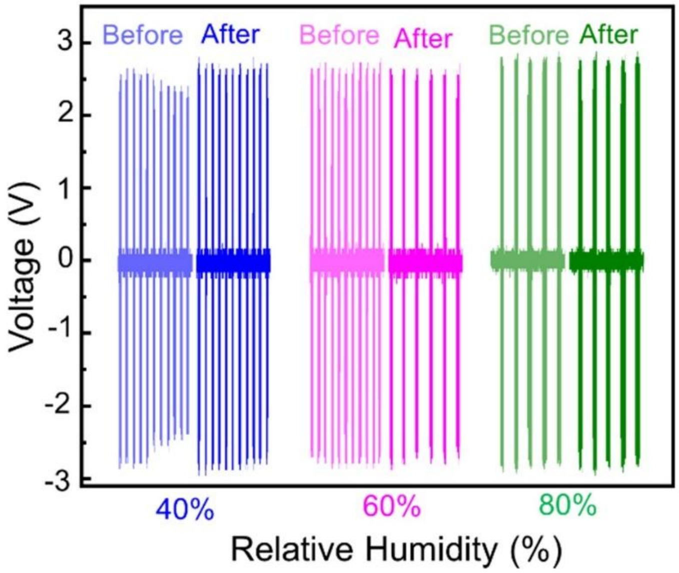

Fig. S11.

Self-healing capability of the E-Skin at different humidity levels, represented by 40%, 60%, and 80% humidity. The bars labeled "Before" and "After" indicate the E-Skin's performance prior to the self-healing process. The results demonstrate that humidity levels do not influence the self-healing capability of the E-Skin.

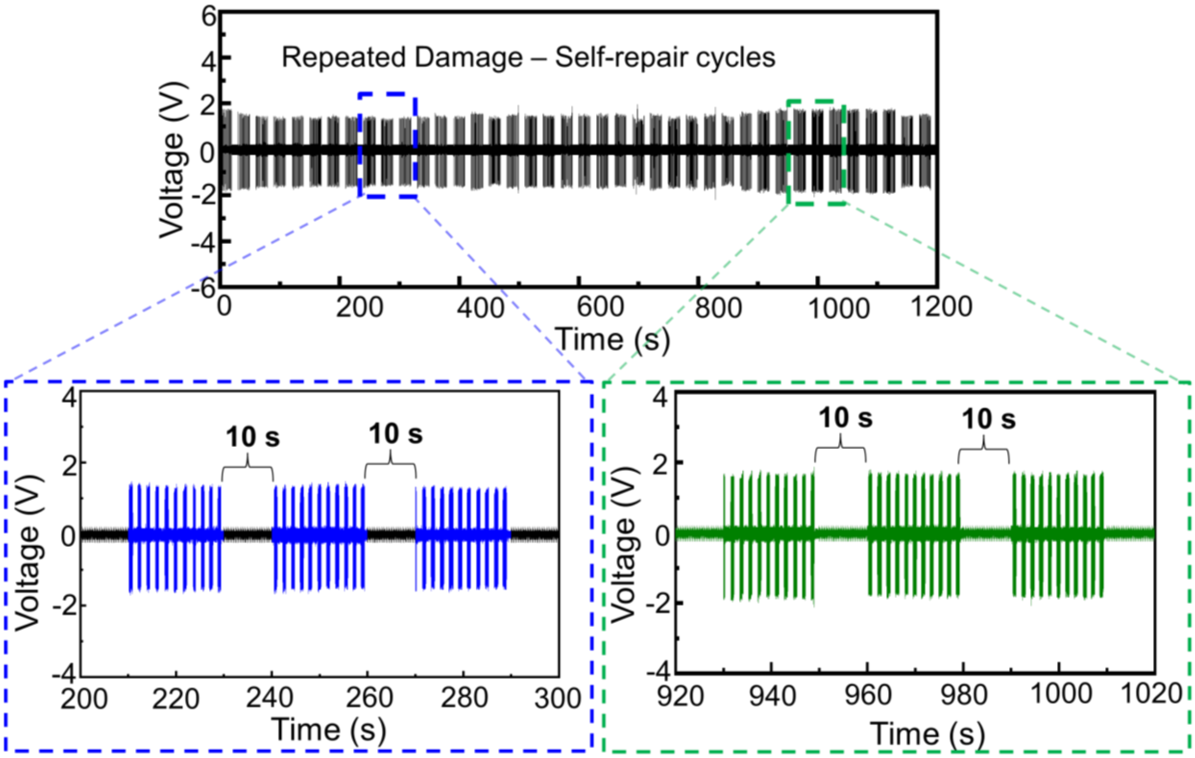

Fig. S12.

Fatigue analysis of the E-Skin throughout repeated cycles of cutting and self-healing. The device underwent up to 40 cycles, with 10-second intervals between each cut and self-healing process, while its electrical output was continuously monitored during touch tests. The results confirm the reliability of the E-Skin's rapid self-healing capability.

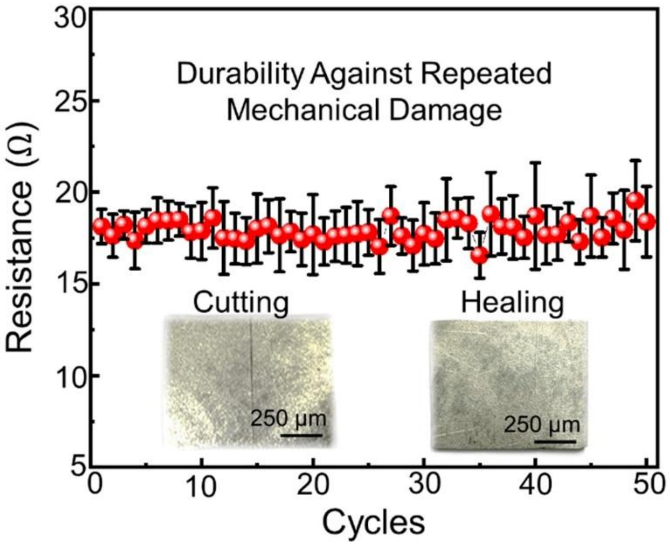

Fig. S13.

Durability of the E-Skin against repeated mechanical damage. Minimal conductivity variation was observed throughout the durability test, with inset photo images highlighting the E-Skin's surface morphology during the self-healing process.

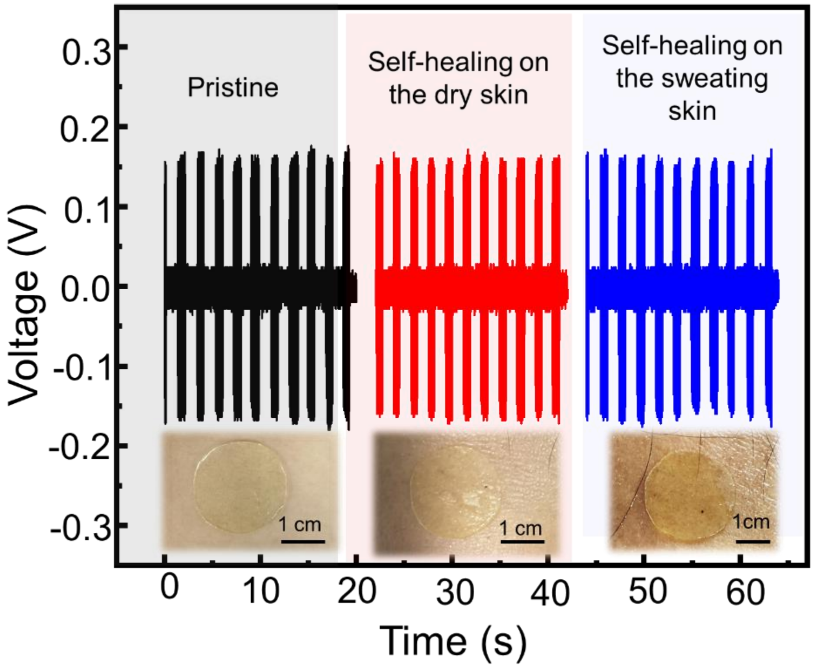

Fig. S14.
Evaluation of the impact of sweating on the self-healing performance of the E-Skin patch. The presence of sweat does not influence the self-healing capabilities, as consistent electrical output is maintained from the self-healed E-Skin patch on sweating skin, comparable to that of the pristine E-Skin patch.

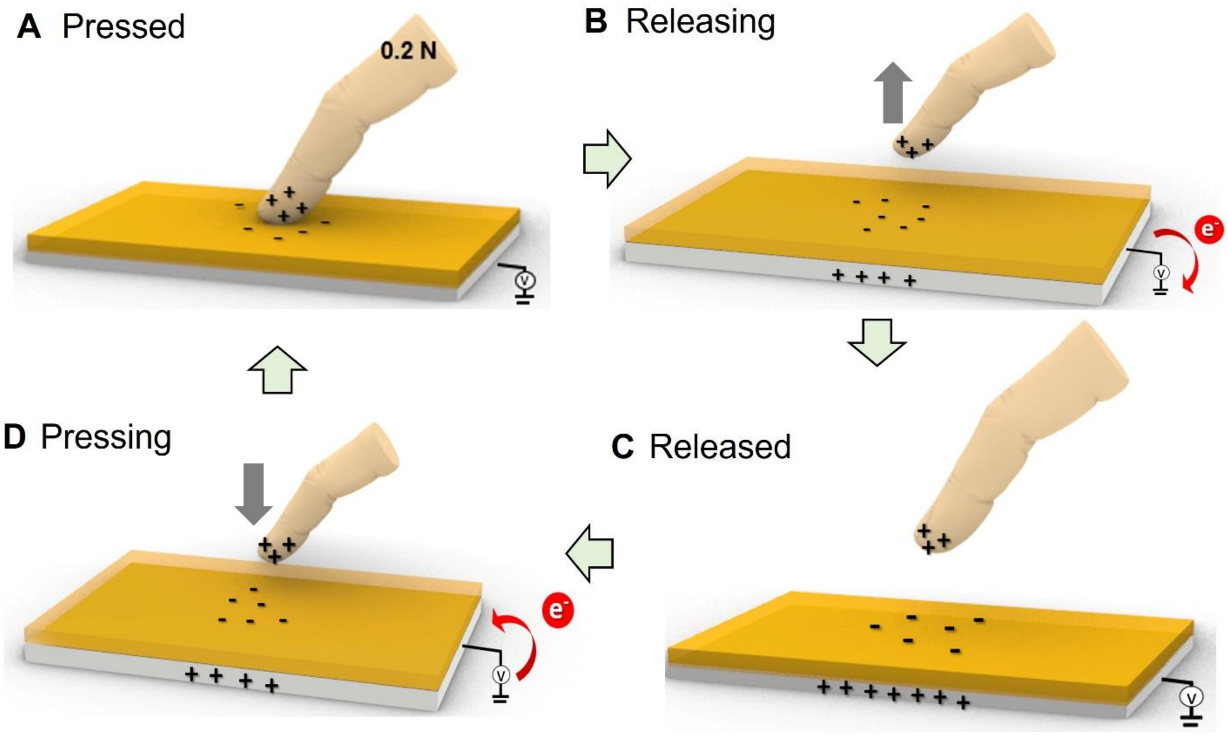

Fig. S15.

Self-powered Sensing Mechanism of the E-Skin. (**A**) Pressed: Contact electrification occurs as the finger presses the E-Skin, causing electron transfer and the accumulation of triboelectric charges. (**B**) Releasing: Removing the finger from the E-Skin initiates charge separation, generating an electric field and inducing a transient current. (**C**) Released: Complete separation maximizes charge distribution and electric field strength, resulting in peak current induction. (**D**) Pressing: The finger approaching the E-Skin redistributes charges, altering the electric field and inducing current due to movement.

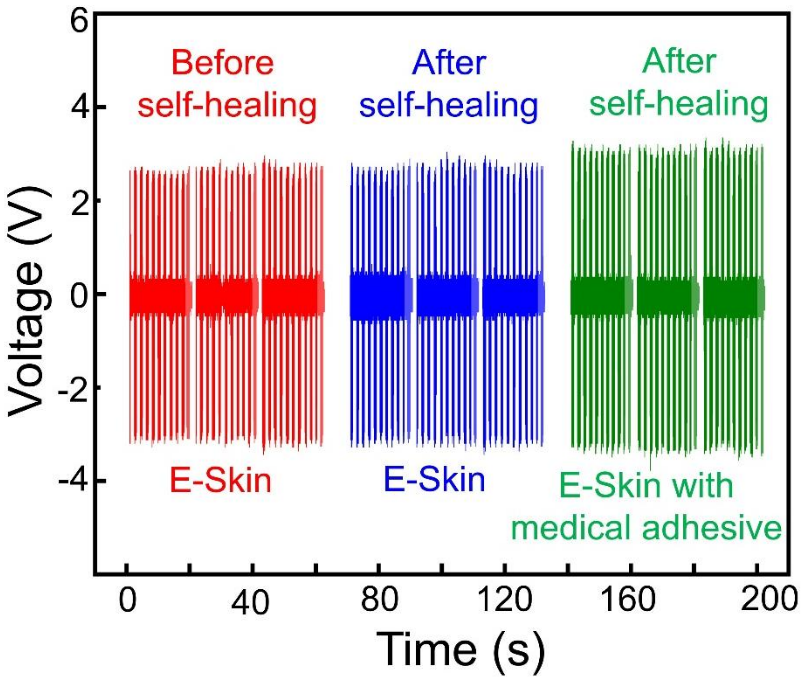

Fig. S16.

Voltage response of the E-Skin before and after self-healing, both with and without medical adhesive. Results show that the application of medical adhesive has minimal impact on self-healing performance.

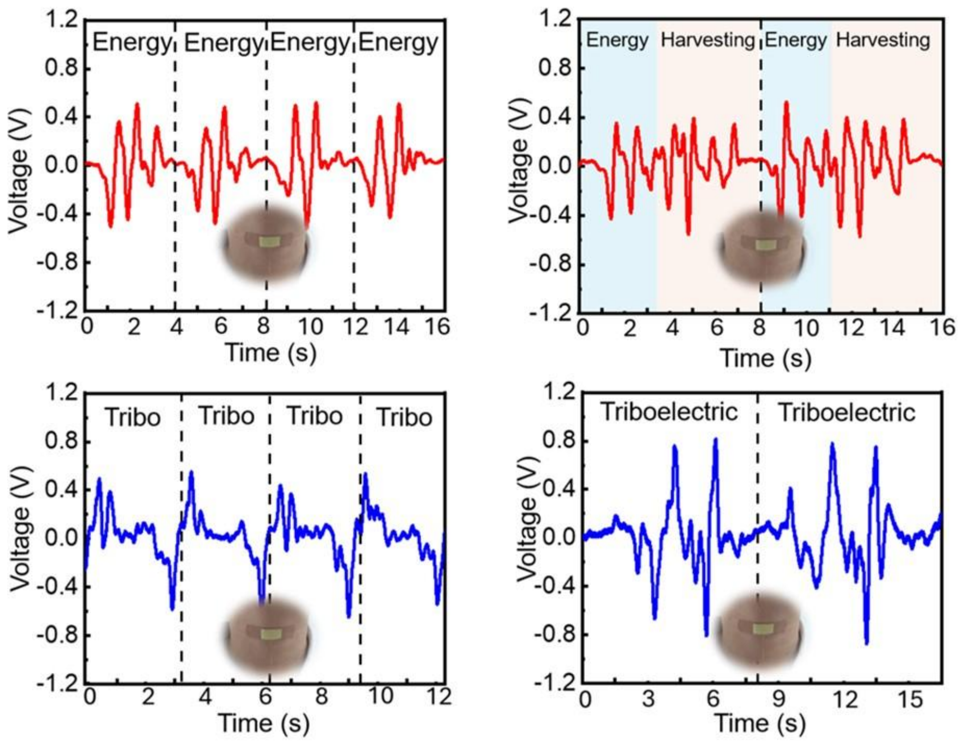

Fig. S17.

Voltage response of the E-Skin mounted on the throat for detecting vocal cord vibrations, facilitating sound and speech pattern recognition. The top row displays the voltage output over time as the subjects spoke the word "energy harvest" repeatedly, showing periodic signals across multiple cycles. The bottom row illustrates the voltage response when the word "triboelectric" was spoken, demonstrating stable signals. Inset images show the placement of the E-Skin on the throat during testing.

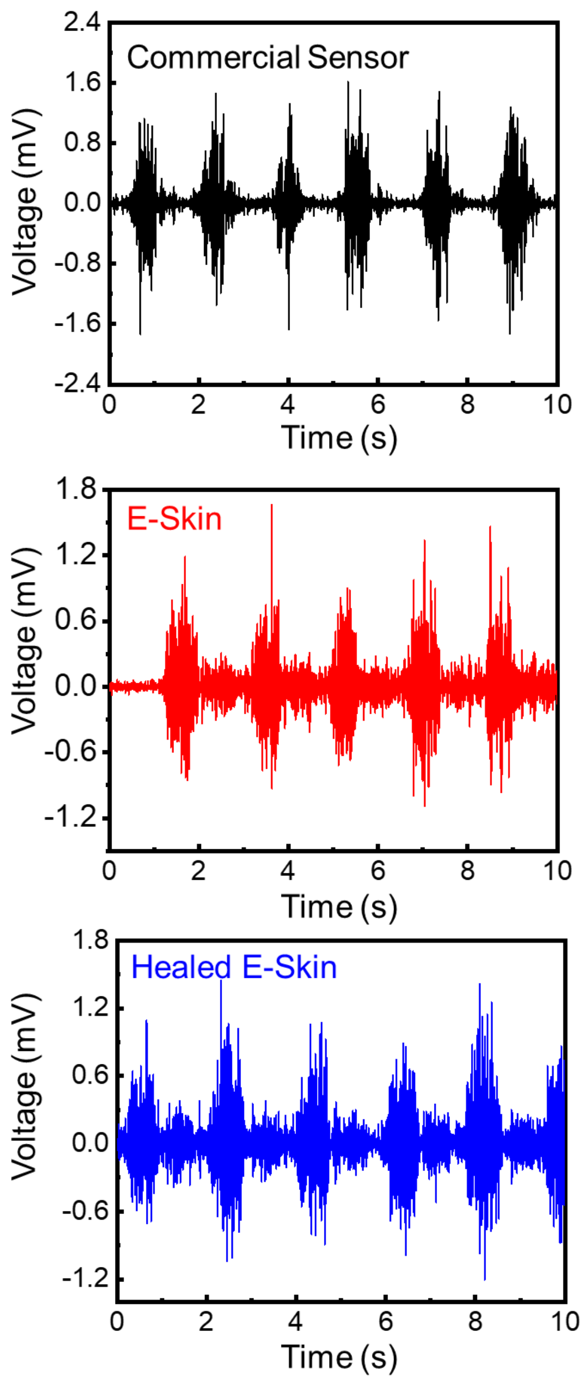

Fig. S18.

sEMG signal responses to fist clenching and unclenching actions, as recorded by a commercial sEMG sensor, E-Skin, and Healed E-Skin. The data demonstrate the healed E-Skin's ability to effectively replicate the signal response of the original E-Skin, indicating successful restoration of sensing performance post-healing.

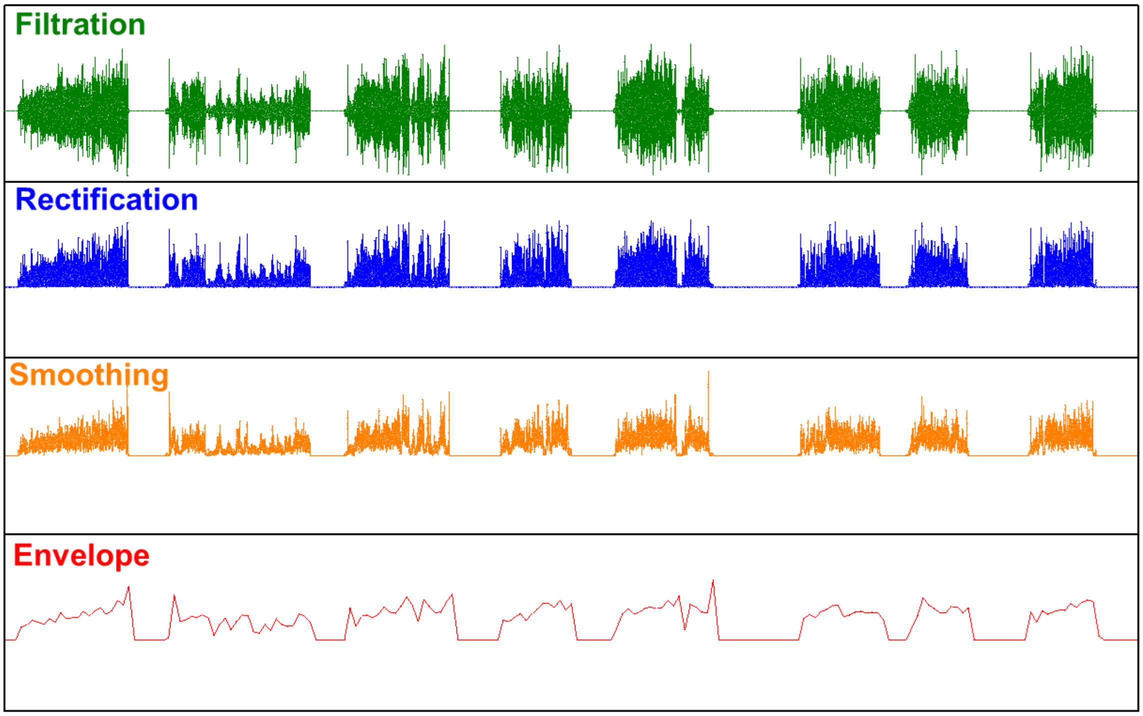

Fig. S19.

sEMG signal process. Green: Filtrated signal, a limit was created for the signal, excluding everything out of it; Blue: Rectified signal, all negative values were transformed in positive ones and added; Orange: the smoothed signal, a linear enveloped was created and the extreme parts of the signal was excluded; Red: The final EMG envelop after all the treatments.

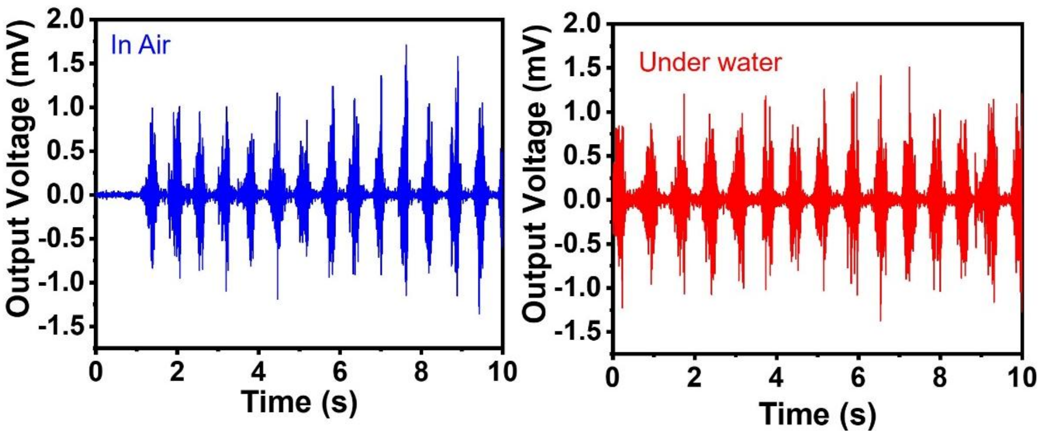

Fig. S20.

sEMG signals recorded by the E-Skin device in different environments, illustrating signal stability and performance. The left panel (blue waveform) shows the sEMG response in air, while the right panel (red waveform) depicts the response underwater. This enhanced visualization highlights the E-Skin's capability to function effectively in both air and aqueous environments.

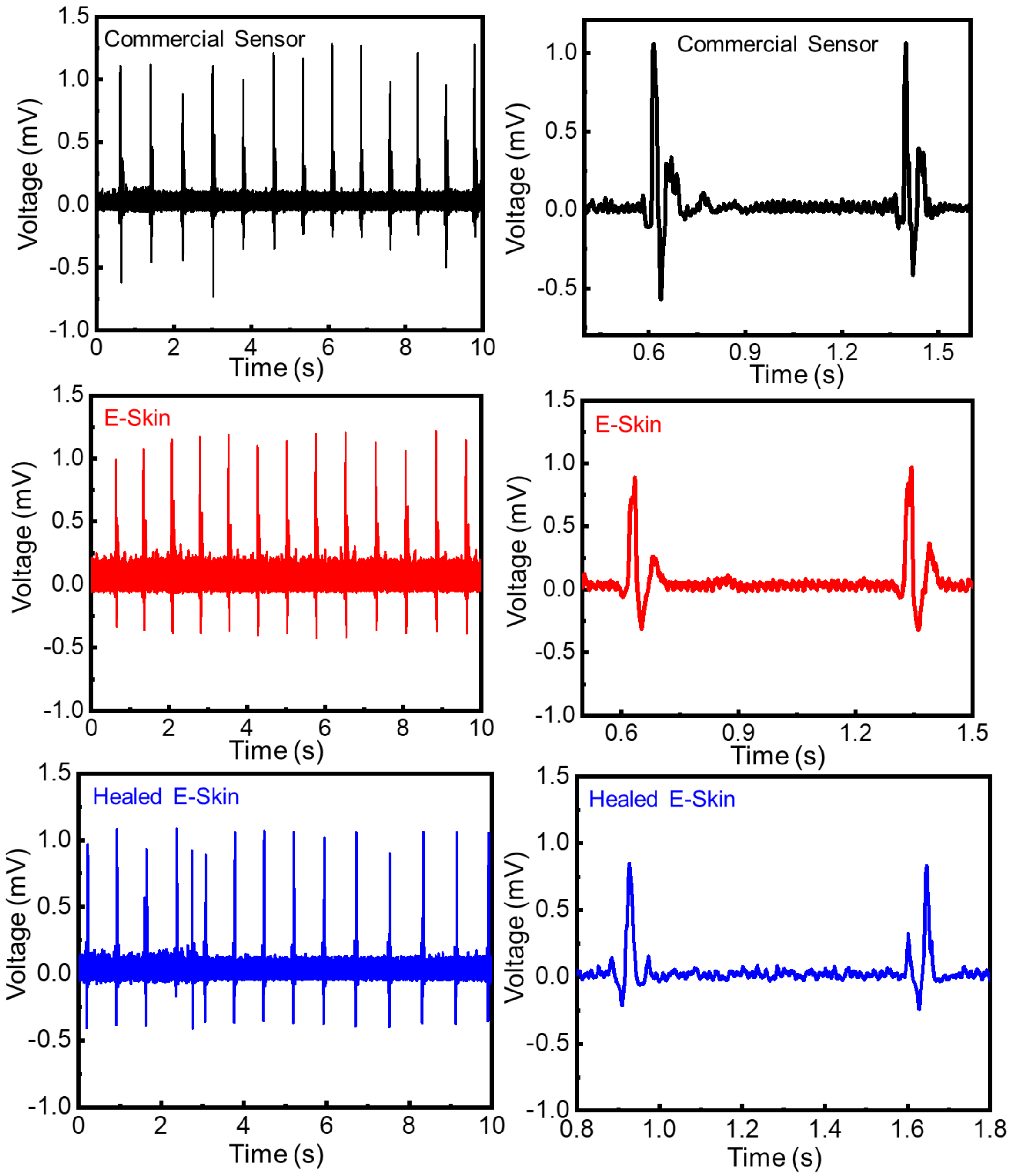

Fig. S21.

Comparison of ECG signal responses during fist clenching and unclenching actions, as recorded by the E-Skin and Healed E-Skin. The data demonstrate that the Healed E-Skin effectively replicates the ECG response patterns of the original E-Skin, indicating preserved sensing capability post-healing.

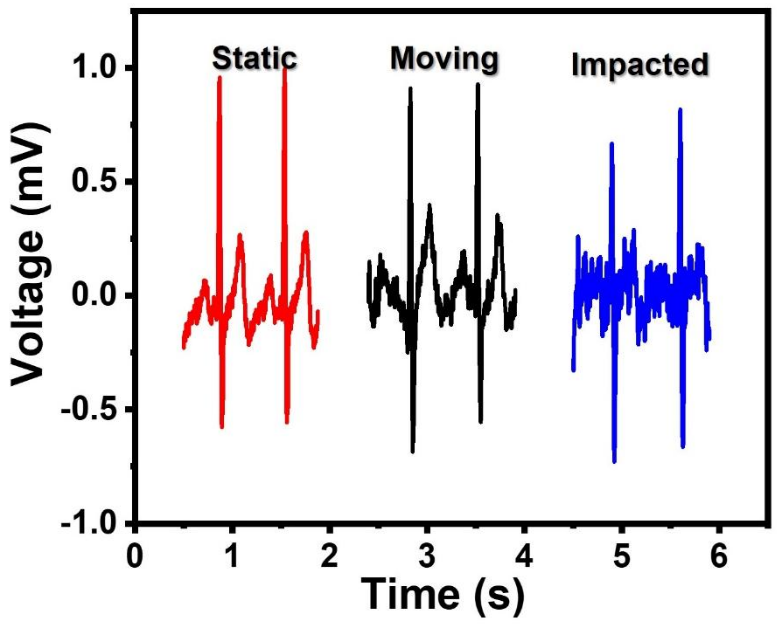

Fig. S22.

Enlarged view of the ECG signals recorded from the E-Skin in water under various conditions: stationary, dynamic, and subjected to impact. The findings illustrate the E-Skin's ability to capture ECG signals reliably in an aqueous environment, regardless of external motion or impact.

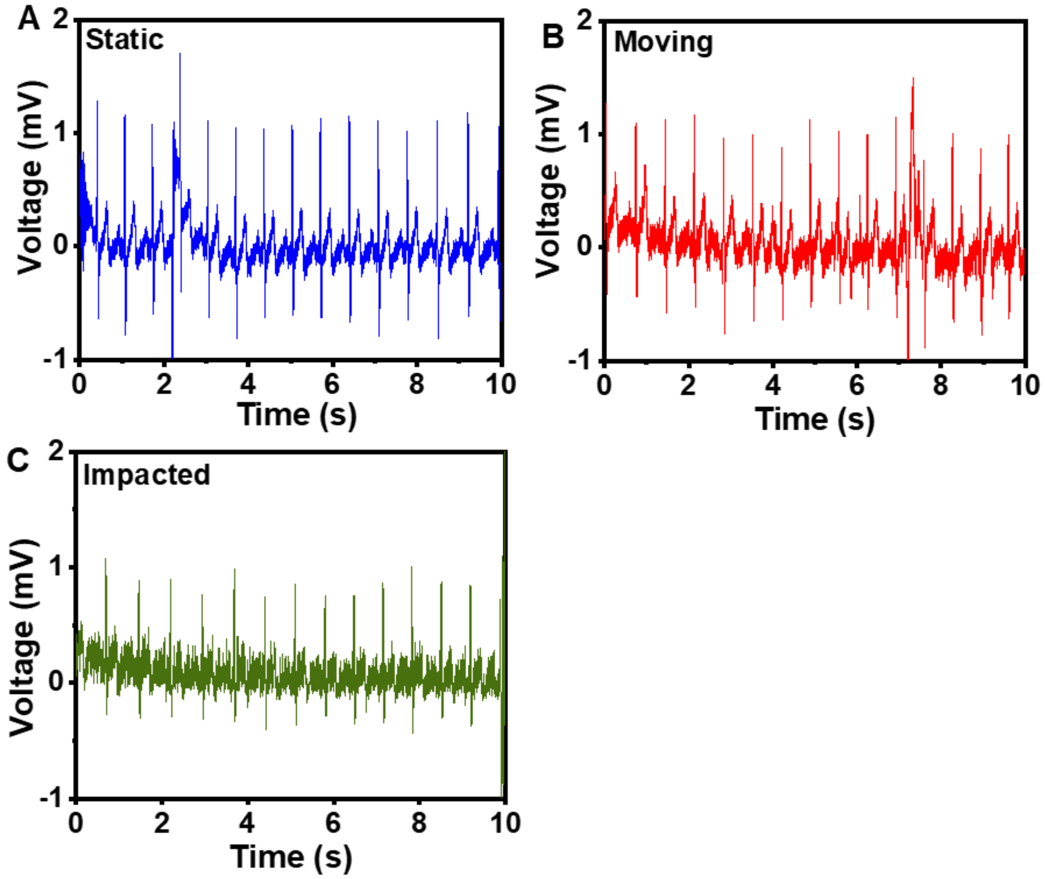

Fig. S23.

ECG signals recorded by the E-Skin in an aqueous environment under different conditions: (A) static, (B) dynamic, and (C) impacted. These recordings illustrate the E-Skin's capability to maintain reliable ECG signal detection across varying environmental conditions.

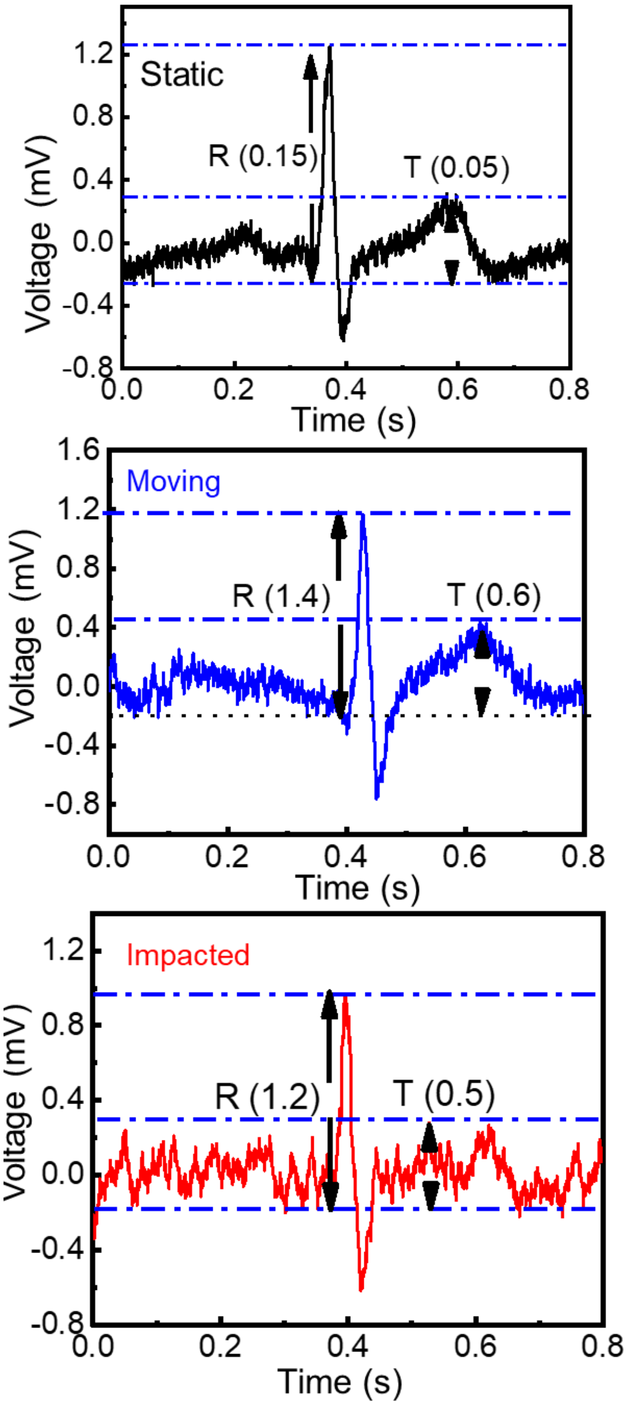

Fig. S24.

Measurement of the T/R ratio from ECG signals by the E-Skin in underwater environments across different conditions: static, moving, and subjected to impact. The data show consistent T/R ratio measurements across conditions, demonstrating the E-Skin's ability to accurately monitor ECG signals in dynamic and impacted states in an aqueous setting.

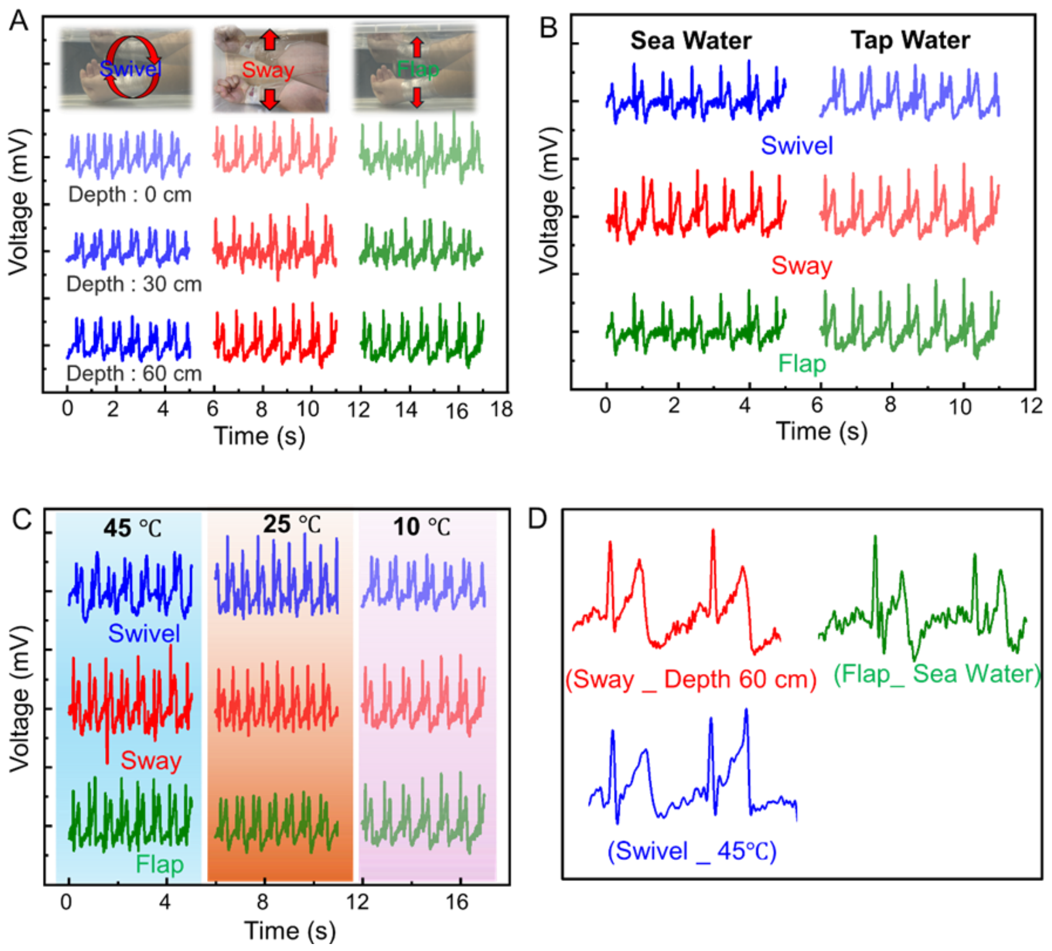

Fig. S25.

Continuous ECG monitoring during dynamic swivel, sway, and flap movements. (**A**) Comparison of ECG si6gnals at varying water depths (0 cm, 30 cm, 60 cm). (**B**) Comparison of ECG signals across different water types (tap water, seawater). (**C**) Comparison of ECG signals at different water temperatures (10°C, 25°C, 45°C). (**D**) Enlarged ECG signal captured during Sway at a depth of 60 cm, Flap in seawater, and Swivel at 45°C.

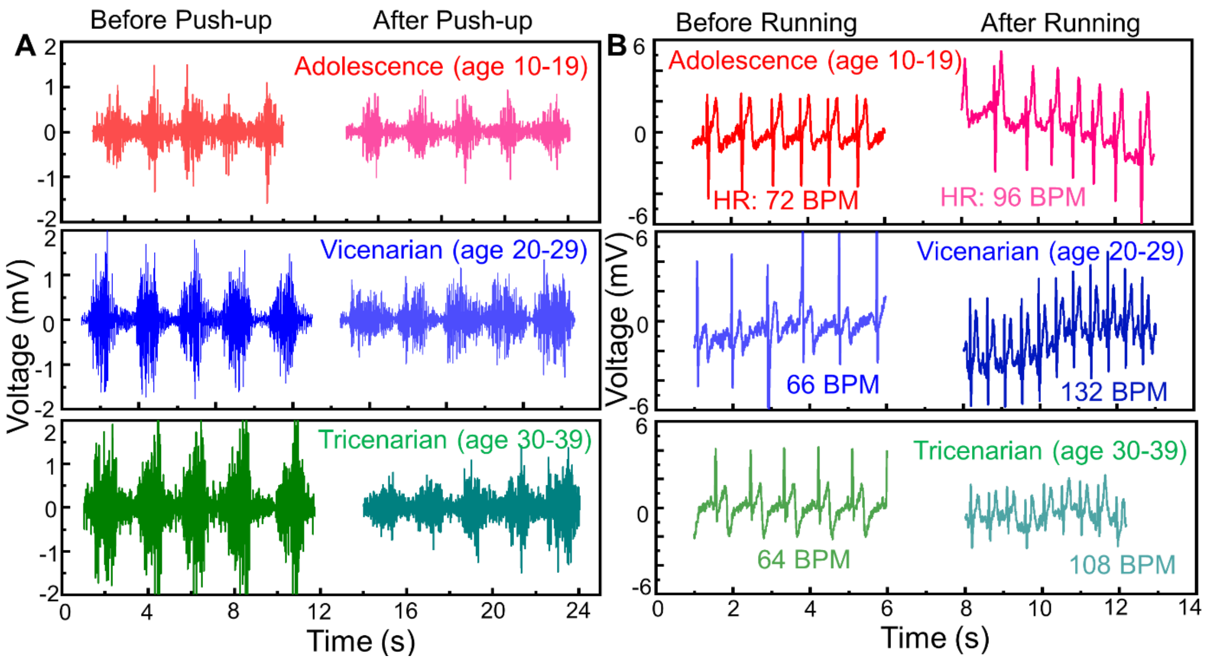

Fig. S26.

A comparison of EMG and ECG measurements in subjects of varying ages, taken before and after exercise. (**A**) EMG comparison before and after performing push-ups. (**B**) ECG comparison before and after running.

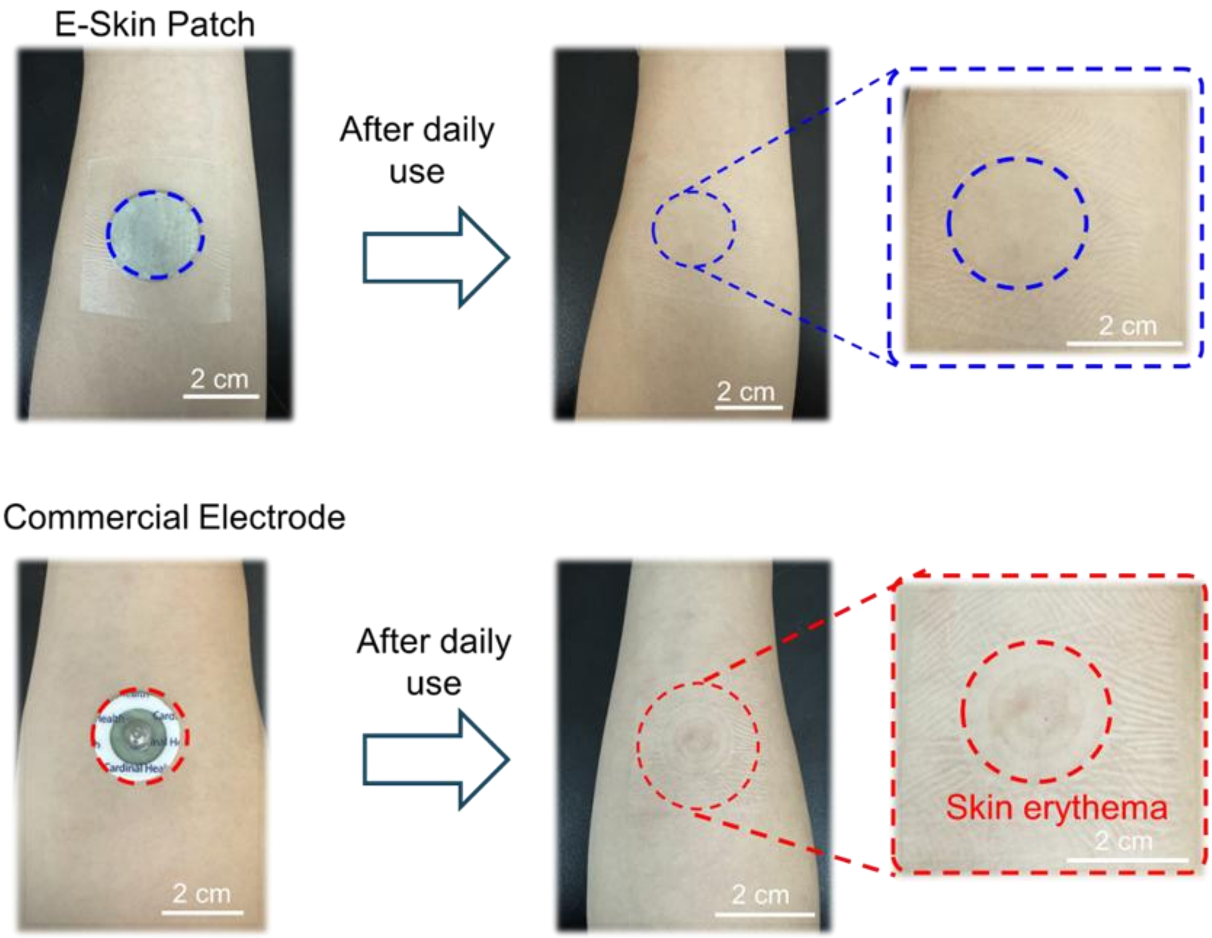

Fig. S27.

Images of the E-Skin patch mounted on the forearm, compared to the commercial EMG electrode. It does not induce significant adverse effects after one day of wearing. By contrast, the skin has obvious erythema after 1 hour of wearing gel electrodes.

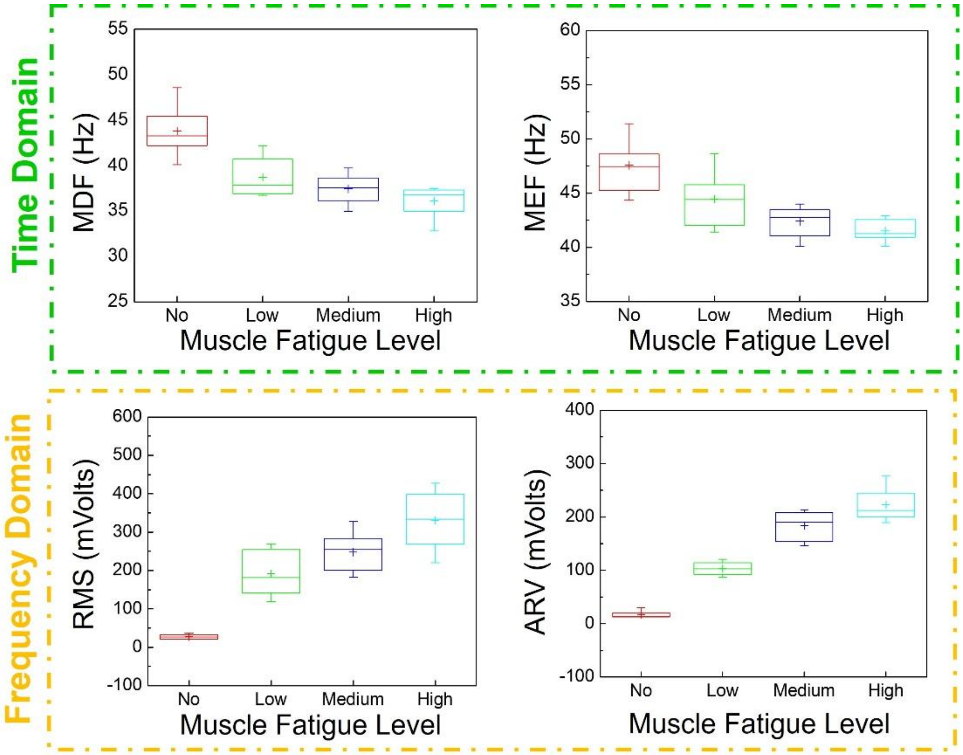

Fig. S28.

Quantification of muscle fatigue levels using E-Skin attached to the biceps. Clear trends in biometrics, including MDF, MEF, ARV, and RMS, were observed during muscle fatigue. Notably, higher ARV and RMS values corresponded to increased muscle fatigue, whereas slightly lower MDF and MPF values were recorded in subjects with elevated fatigue levels. These findings are consistent with previous reports.

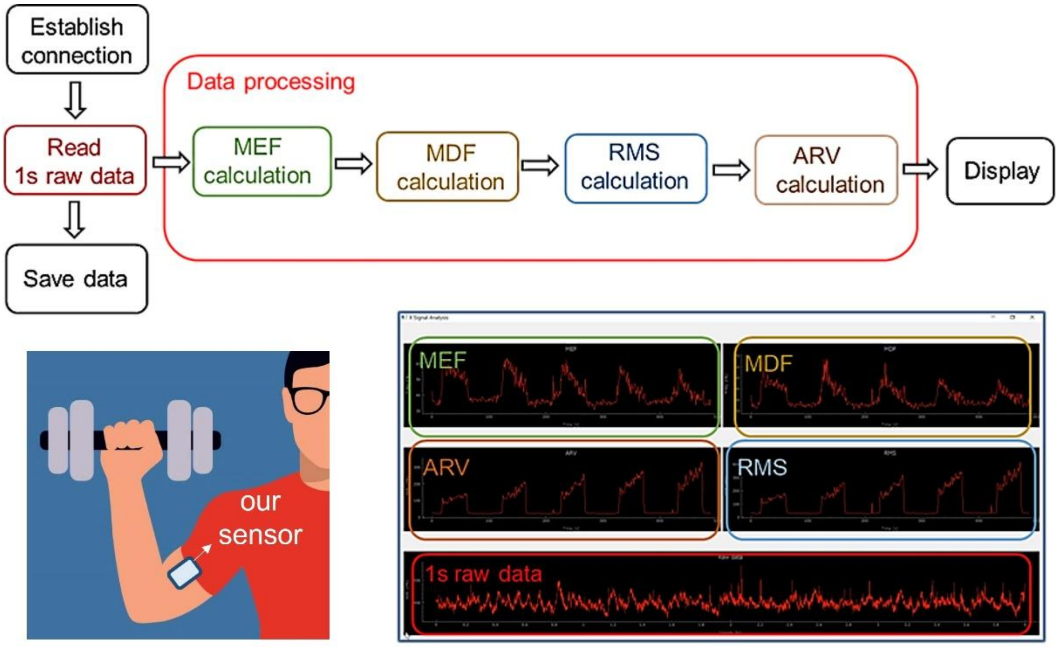

Fig. S29.

Real-time graphic user-interface (GUI) for digital front end (GFE). The GUI workflow includes establishing a connection, reading 1-second intervals of raw data, and performing data processing steps such as Mean Frequency (MEF), Median Frequency (MDF), Root Mean Square (RMS), and Average Rectified Value (ARV) calculations. Processed data is then displayed in real-time. The lower left panel illustrates the E-Skin placement on the arm during exercise, while the lower right panel displays real-time output graphs of MEF, MDF, RMS, ARV, and raw data.

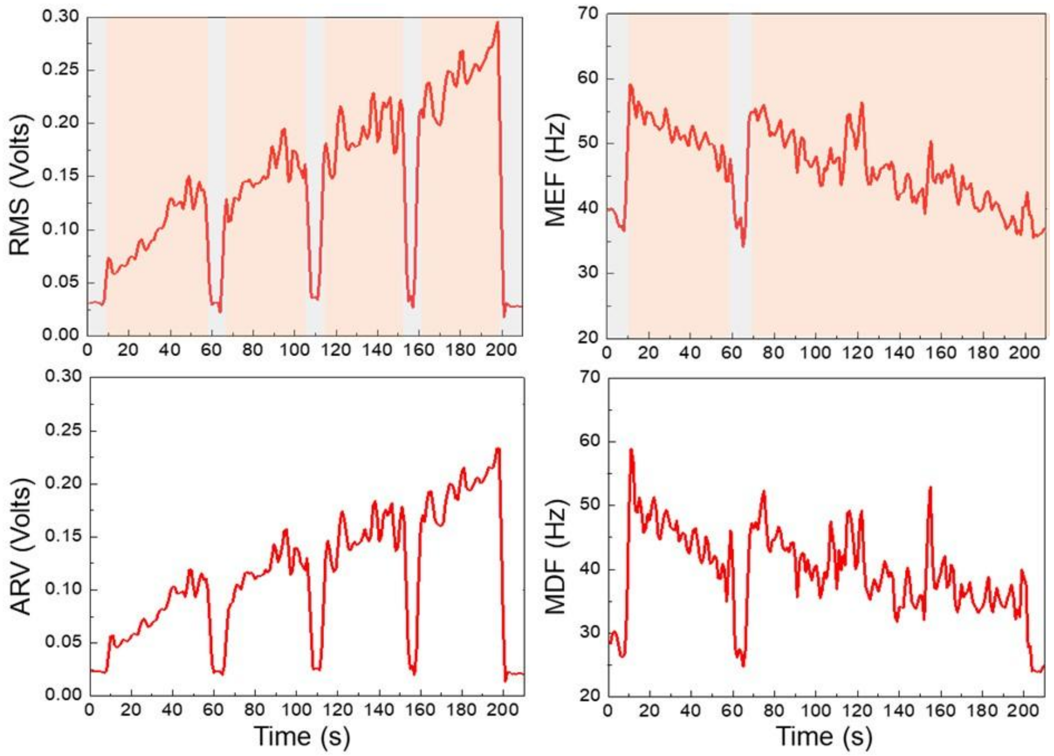

Fig. S30.

Real-time visualization of comprehensive biometric data for monitoring muscle fatigue. These visualizations, including RMS, MEF, ARV, and MDF measurements, demonstrate the E-Skin's capability to monitor muscle performance and fatigue dynamically.

Table S1.

Overview of electrode materials used in recent E-Skin applications

Flexible Electrode	Stretchability (%)	Durability (cycles)	Bendability (cycles@radius)	R_s (Ω/sq)	Ref**.**
AgNWs	**900**	**30000**	**50000@1mm**	**18**	**This work**
Graphene	150	5000	100@90°	1500	*(22)*
CNT/graphene	150	700	N/A	110	*(23)*
Metal NW/CNT	200	50	N/A	9000	*(24)*
MXene	N/A	N/A	1000@5mm	200	*(25)*

Table S2.

Comparative analysis of existing devices and our E-Skin patch

Ref.	Self-healing Time (s)	Self-healing capability (%)	Self-healing Temperature (℃)
**This work**	**10**	**81**	**Room Temperature (25 ℃)**
(44)	43200	100	37
(45)	600	100	70
(46)	72000	10	40
(47)	3600	71.5	85
(48)	7200	90	75
(49)	600	86	20
(50)	10800	94	80
(51)	10800	100	25
(52)	64800	90	40
(53)	1200	100	40
(54)	6000	60	25
(55)	86400	95	25
(56)	86400	90.3	25

Table S3.

EMG-based metrics that reflect muscle fatigue

Domain	Parameters	Equation	Note
Time Domain	Mean Absolute Value (MAV)	$MAV = \frac{\sum_{i=1}^{N} \lvert EMG_{\mathrm{i}} \rvert}{N}$	Average absolute value of EMG amplitude
	Root Mean Square Level (RMS)	$RMS = \sqrt{\frac{\sum_{i=1}^{N} EMG^2}{N}}$	Norm 2 of the EMG amplitude divided by the square root of the number of samples
Frequency Domain	Average Frequency (MEF)	Power (EMG, f [0HZ, 250HZ])	Power of the EMG the signal in the frequency domain in the interval [0HZ, 250HZ]
	Median Frequency (MDF)	Power (EMG, f [0HZ, MDF])<br><br>Power (EMG, f [MDF, 250HZ])	Half of the signal power is distributed in the frequencies less than MDF
Extracted EMG signal metrics in time and frequency domain			

Table S4.

Characteristics of the subjects

*Table 1. Characteristics of the subjects.**

Characteristic	Subjects to quantify level of muscle fatigue (physical/subjective)
Age --- yr	24 ± 3
Gender --- no. / total no.(%)	
Male	16 (76)
Female	5 (24)
Height --- cm	174 ± 4
Weight --- kg	72 ± 13
Median body-mass index (IQR) §	25 (21-26)
Exercise Frequency--- no.(%)	
0 times per week	4 (19)
1-3 times per week	16 (76)
>3 times per week	1 (5)
Highest Educational Level--- no. (%) ¥	
Less than Master Degree	10 (47)
Advanced Degree (Including Master Degree)	11 (53)
Private medical insurance --- no./total no. (%)	20/21 (95)
Feeling fatigue in daily life – no.(%)	
Yes	18 (86)
No	3 (14)

* Plus-minus values are means ± SD. IQR denotes interquartile range.

§ Body-mass index is the weight in kilograms divided by the square of the height in meters.

¥ Data for the highest level of education completed by subjects.

Table S5.

Evaluation of muscle fatigue

Rate of Perceived Exertion Chart (Modified Borg Scale)	
10	**Maximum Effort Intensity**<br>You cannot keep this pace for more than 1 minute, feels almost impossible to keep going<br>Completely out of breath<br>Unable to talk
9	**Very Hard Intensity**<br>Very difficult to maintain intensity, you are wondering how long you can go like this<br>Breathing really hard<br>Can speak only 1 word at a time
7-8	**Vigorous Intensity**<br>Almost feels uncomfortable<br>Breathing pretty hard now<br>Can speak a sentence or two but requires maximum effort
4-6	**Moderate Intensity**<br>Feels like you can maintain this intensity for hours<br>Breathing more heavily<br>Can carry on a short conversation but requires more effort
2-3	**Light Intensity**<br>Feels like you can maintain this intensity for hours (warm up, cool down, and stretching)<br>Easy to breath<br>Easy to carry on conversation
1	**Very Light Intensity**<br>Any activity between sleeping and light activity (reading, driving, folding laundry, etc.)<br>Breathing not changed<br>Effortless to carry on a conversation

Movie S1.
Rapid Self-Healing in E-Skin Enables Immediate LED Illumination After Damage

Movie S2.
On-Body Verification of E-Skin's Triboelectric Force Detection After Self-Healing

Movie S3.
Dynamic Self-Healing of E-Skin Compared to PDMS Under Ambient Conditions

Movie S4.
E-Skin's Temperature Tolerance and Waterproof Properties in Action

Movie S5.
Real-Time Monitoring of Joint Movements and Physiological Signals (2X speed)

Movie S6.
Forearm-Mounted E-Skin Records sEMG Signals During Muscle Clenching and Relaxing

Movie S7.
E-Skin Tracks Muscle Activity Underwater, Retaining Conductive and Adhesive Properties

Movie S8.
Performance in Underwater ECG Monitoring Across Static, Motion, and Water Flow Conditions

Movie S9.
Real-Time EMG Visualization Through Graphic User Interface
